# The neuroprotective mechanism of lithium after ischaemic stroke

**DOI:** 10.1038/s42003-022-03051-2

**Published:** 2022-02-03

**Authors:** Beina Chen, Manman Zhang, Ming Ji, Dianjun Zhang, Binjie Chen, Wenliang Gong, Xinyu Li, Yuefei Zhou, Chengyi Dong, Gehua Wen, Xiaoni Zhan, Xiafang Wu, Huiya Yuan, Enyu Xu, Maosheng Xia, Alexei Verkhratsky, Baoman Li

**Affiliations:** 1 Department of Forensic Analytical Toxicology, School of Forensic Medicine, China Medical University, Shenyang, China; 2 Department of Orthopaedics, The First Hospital, China Medical University, Shenyang, China; 3 Faculty of Biology, Medicine and Health, The University of Manchester, Manchester, UK; 4 Achucarro Center for Neuroscience, IKERBASQUE, 48011 Bilbao, Spain; 5 Sechenov First Moscow State Medical University, Moscow, Russia

**Keywords:** Cellular neuroscience, Stroke, Cell death in the nervous system

## Abstract

Stroke causes degeneration and death of neurones leading to the loss of motor function and frequent occurrence of cognitive impairment and depression. Lithium (Li^+^), the archetypal mood stabiliser, is neuroprotective in animal models of stroke, albeit underlying mechanisms remain unknown. We discover that Li^+^ inhibits activation of nucleotide-binding oligomerisation domain-like receptor family pyrin domain-containing 3 (NLRP3) inflammasomes in the middle cerebral artery occlusion (MCAO) stroke model in mice. This action of Li^+^ is mediated by two signalling pathways of AKT/GSK3β/β-catenin and AKT/FoxO3a/β-catenin which converge in suppressing the production of reactive oxygen species (ROS). Using immunocytochemstry, MRI imaging, and cell sorting with subsequent mRNA and protein quantification, we demonstrate that Li^+^ decreases the infarct volume, improves motor function, and alleviates associated cognitive and depressive impairments. In conclusion, this study reveals molecular mechanisms of Li^+^ neuroprotection during brain ischaemia, thus providing the theoretical background to extend clinical applications of Li^+^ for treatment of ischemic stroke.

## Abstract (web summary)

Using the MCAO stroke mouse model, Chen et al. identify pathways that underlie the neuroprotective properties of lithium by using inhibitors of AKT/GSK3β/β-catenin and AKT/FoxO3a/β-catenin signaling. They report that the inhibitors reverse the protective effects of lithium thus conclude that both pathways are key for lithium-mediated neuroprotection.

## Introduction

Ischaemic stroke is a severe life-threatening disease, accounting for 75–80% of all strokes, which have been the second leading cause of death worldwide^1–3^. Several treatments aimed to restore blood flow (reperfusion), such as intravenous thrombolysis and thrombectomy, have been developed in recent decades^4^. Nevertheless, progressive degeneration and death of neurones responsible for loss of brain function remains difficult-to-solve issue for treatment and rehabilitation. Cognitive impairment and depression are common stroke complications^5,6^, with the occurrence rate close to 30%^7,8^. Improving neurological outcome after an ischaemic stroke is a major societal priority, which has attracted much attention of clinical and basic research, government funding agencies, and industry. Majority of stroke neuroprotectants effective in rodents research have failed to translate into clinical practice^9,10^. Repurposing market-approved drugs for treatment of the cerebral ischaemia therefore represents a viable strategy.

Lithium (Li^+^) was first used in clinical practice for dissolving urinary calculi^11^; whereas first psychiatric usage of Li^+^ as anticonvulsant, hypnotic and anti-mania drug has been reported in 1870s^12,13^. The modern era of Li^+^ usage in psychiatry begun in1952 when Li^+^ therapy was considered to be a useful alternative to then all-popular electroconvulsive therapy^14,15^. Therapy with Li^+^ is effective in treatment of acute mania and acute depression as well as prevention of recurrent mania and depression^16,17^. In recent two decades, Li^+^ has been reported to exert neuroprotection in animal models of stroke, neurodegenerative diseases, spinal cord injury and other impairments of the central nervous system^18^. Randomised clinical trial of the effects of Li^+^ on post-stroke patients demostrtrated enhanced motor recovery following an early treatment with a low dose of lithium carbonate^19^. The effective and safe dose range for Li^+^ in serum is narrow ranging between 0.6 and 1.0 mM; at concentrations exceeding 1.5 mM Li^+^ is toxic. Serum Li^+^ levels of 1.5–2.0 mM produce mild and reversible toxicity at kidney, liver, heart, and secretory glands. Serum levels above 2 mM may be associated with neurological symptoms, including cerebellar dysfunction^17^. Narrow safety range limits the neuroprotective usage of Li^+^ for ischaemic stroke^20^, as well as for associated dementia or depression symptoms^21–23^.

Mounting evidence has demonstrated that NLRP3 (nucleotide-binding oligomerisation domain-like receptor family pyrin domain-containing 3) inflammasome plays a prominent role in the pathogenesis and progression of ischaemic stroke^24–26^. Many aspects of the biology of NLRP3 inflammasome in stroke remain, however, unknown. While numerous activators of the NLRP3 inflammasome have been identified, the underlying mechanism of activation has not been fully elucidated. Existing hypotheses include interaction with reactive oxygen species (ROS), lysosomal rupture, and potassium efflux^27,28^. Accumulation of ROS and oxidative stress contribute to brain injury after ischaemic stroke, with suppression of ROS overproduction being considered as a therapeutic strategy^29^.

The NLRP3 inflammasome is a multiprotein complex, assembled from the adaptor protein apoptosis-associated speck-like protein containing a CARD (ASC) and inflammatory caspase-1 (cysteine-dependent aspartate-directed protease 1). The major function of NLRP3 inflammasome is to recognise a wide range of danger signals associated with lesions to nervous tissue. Through recognition of damage-associated molecular patterns, the NLRP3 inflammasome, with its ASC and pro-caspase-1 components, promotes the activation of caspase-1 and the processing of cytoplasmic targets, including IL-1β and IL-18^30^. During this process, gasdermin D (GSDMD), a physiological substrate of the canonical inflammasome pathway and pyroptosis executor is recruited to the NLRP3 inflammasome and cleaved by pro-caspase-1 and matured caspase-1. The N-terminal fragment of caspase-1-cleaved GSDMD approaches the plasma membrane and oligomerises to form plasma membrane pores, thus triggering pyroptosis and promoting maturation and release of IL-1β^31,32^. Accumulating evidence suggests that NLRP3 inflammasome plays an essential role in the pathogenesis of ischaemic stroke^23–25^, however, the cell-specific expression and distribution of NLRP3 inflammasome is not fully characterised. There are indications for NLRP3 expression in microglia and endothelial cells but not in neurones^33^, as well as data showing an increase in NLRP3 inflammasome proteins, IL-1β and IL-18 in the ipsilateral neurones of cerebral ischaemic and reperfused mice as well as in neurones from the postmortem stroke patients brain tissue^34^.

## Results

### Li^+^ inhibits activation of NLRP3 inflammasome following ischaemic injury

**Fig. 1:**
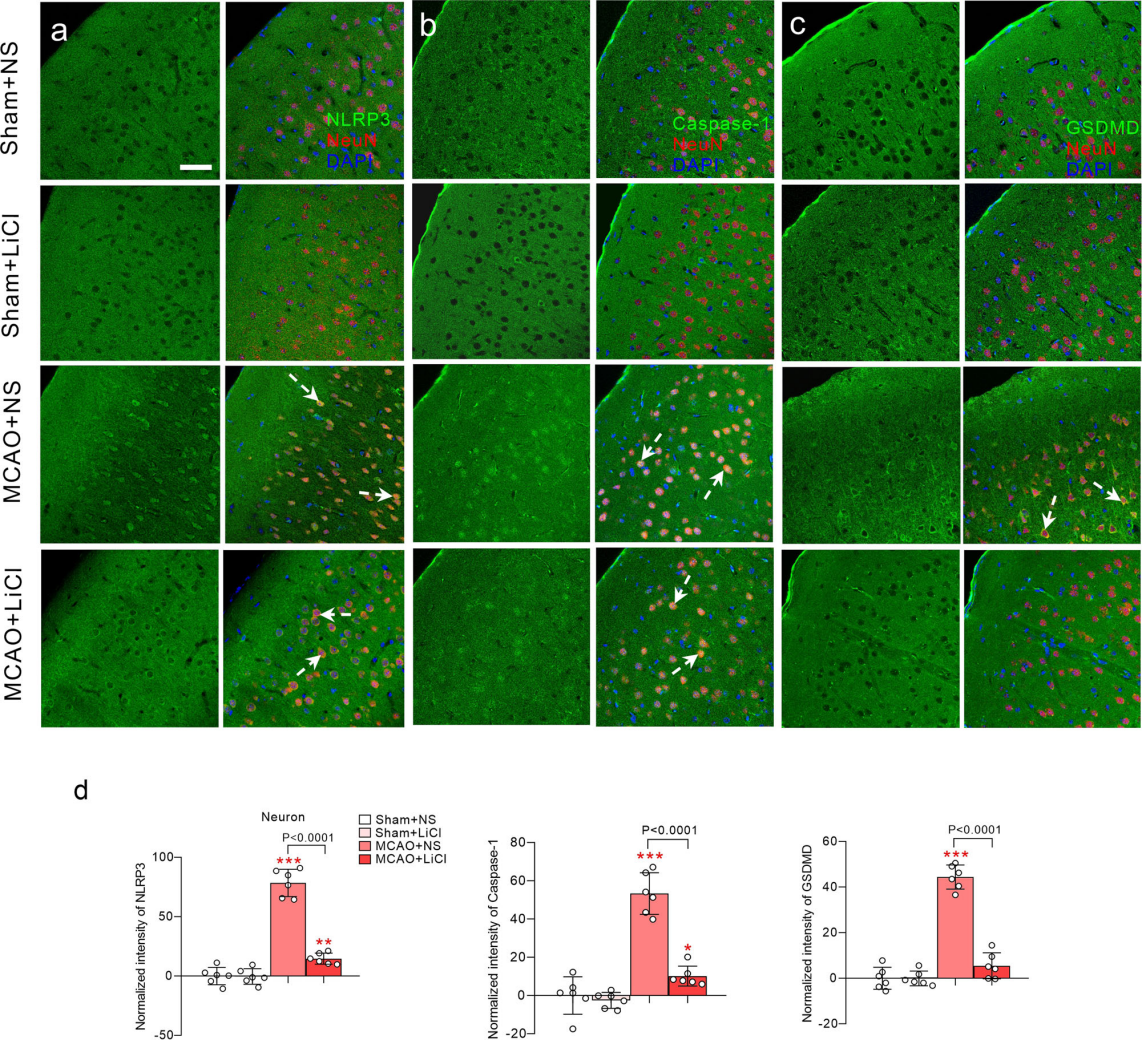
LiCl suppresses neuronal activation of NLRP3 inflammasome and the pyroptosis related GSDMD induced by MCAO. **a**–**c** Green immunofluorescence of NLRP3 (**a**), caspase-1 (**b**) and GSDMD (**c**) co-stained with neuronal marker NeuN (red) and nucleus marker DAPI (blue) in the injured ipsilateral cortex. Scale bar 50 μm. **d** Immunofluorescence intensities of NLRP3, caspase-1 and GSDMD in cortical neurones normalised to sham-NS group. The immunofluorescence intensities were normalised to control group and plotted as mean ± SD. *N* = 6 per group. One-way ANOVA for comparisons including more than two groups; unpaired two-tailed *t*-test for two group comparisons. *p < 0.05, **p < 0.01, ***p < 0.001.

**Fig. 2:**
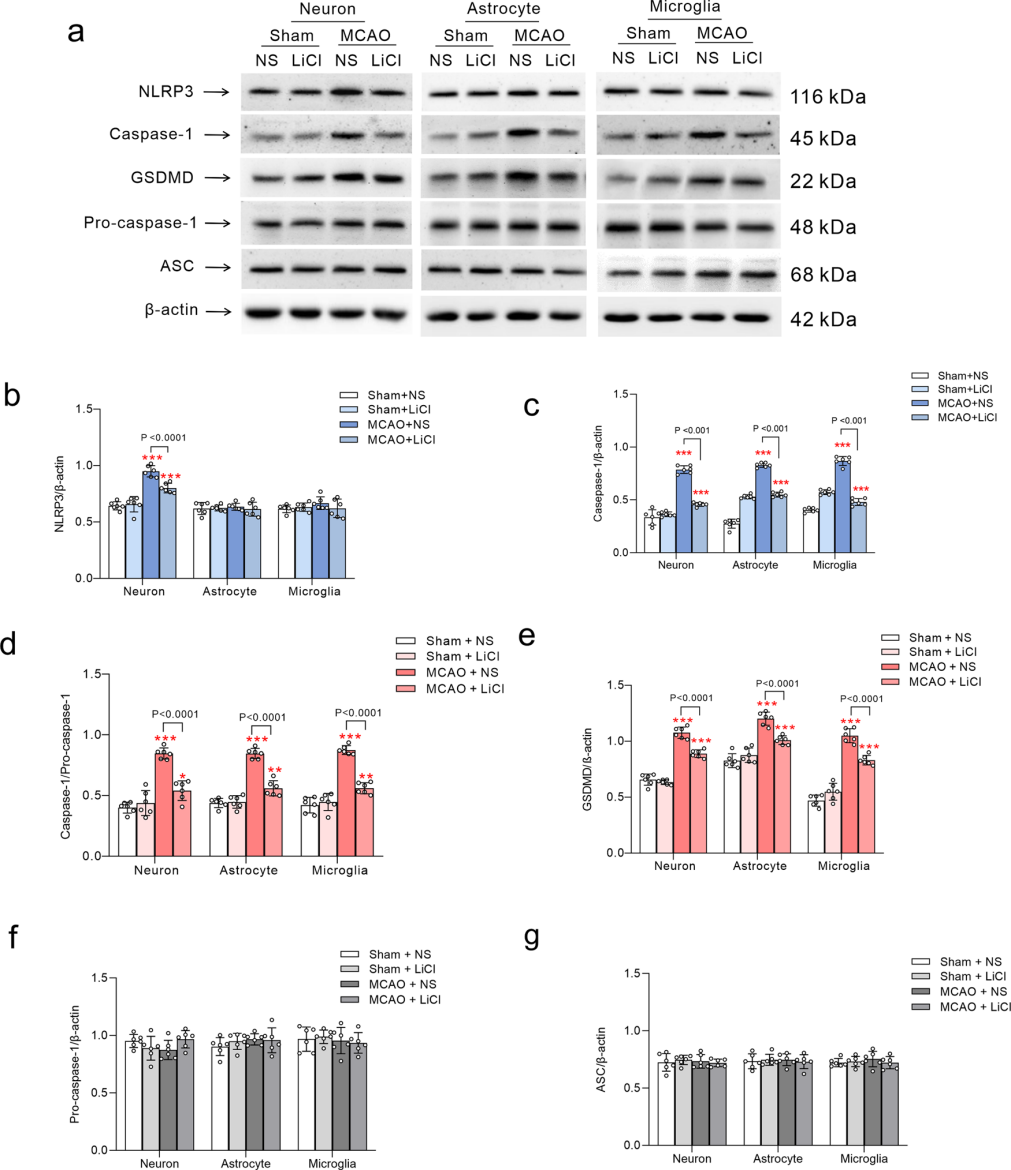
Li^+^ suppresses expressions of NLRP3 and GSDMD induced by ischaemia-reperfusion. Cortices of Thy1-YFP and GFAP-GFP transgenic mice were used for isolating and FACS sorting neurones and astrocytes, respectively, for FACS sorting of microglia the cells from cortex of wild-type mice were labelled with Iba1. **a** The representative protein bands of NRLP3, caspase-1, GSDMD, pro-caspase-1, ASC and β-actin (the house-keeping protein) in neurones, astrocytes and microglia. **b**–**g** Normalised intensities of NLRP3, caspase-1, GSDMD, pro-caspase-1 and ASC by β-actin and the ratio of caspase-1 and pro-caspase-1 are presented as mean ± SD. *N* = 6 per group. One-way ANOVA for comparisons including more than two groups; unpaired two-tailed *t*-test for two group comparisons. *p < 0.05, **p < 0.01, ***p < 0.001.

We modelled ischaemic injury by 1 h middle cerebral-artery occlusion (MCAO) followed with reperfusion. The following experimental groups were established: sham + normal solution (NS) group (control), sham + lithium chloride group (LiCl), MCAO + NS group (MCAO) and MCAO + LiCl group. The details of experimental design are shown in Supplementary Fig. 1. We intraperitoneally (i.p.) injected LiCl at 1 mmol/kg/day for 5 days, which corresponds to serum Li^+^ concentration of ~0.8 mM and in cerebral tissue concentration of ~0.5 mM^35^. When comparing with control group, the immunofluorescence of NLRP3 increased specifically in neurones of ischaemic ipsilateral cortex (*p* < 0.001; Fig. 1a–d); we did not detect any increase in NLRP3 immunofluorescence in astrocytes and microglia (Supplementary Figs. 2d and 3d). An increase in NLRP3 in neurones of MACO-subjected cerebral cortex was effectively suppressed by administration of LiCl at 1 mmol/kg/day (*p* < 0.001; Fig. 1d). The immunofluorescence of caspase-1 and gasdermin D (GSDMD) were both increased by ischaemia-reperfusion in cortical neurones, astrocytes and microglia (*p* < 0.001; Fig. 1d, Supplementary Figs. 2d and  3d). Treatment with Li^+^ significantly decreased ischaemia-induced elevation in immunofluorescence of caspase-1 and gasdermin D (GSDMD) in neurones, astrocytes and microglia as compared with MCAO group (*p* < 0.001; Fig. 1b-d, Supplementary Figs. 2b–d and 3b–d). In summary, ischaemia-reperfusion increased NLRP3 only in neurones, whereas caspase-1 and GSDMD were increased also in astrocytes and in microglia. We further quantified proteins in the populations of neurones (FACS sorted from Thy1-YFP transgenic mice), astrocytes (from GFAP-GFP transgenic mice) and microglia (FACS sorted Iba-1-positive cells of wild-type mice). As in our previous reports^36^, the specificity of the sorted cells were identified by reverse transcription–polymerase chain reaction (RT-PCR) with cellular-specific genes, shown in Supplementary Fig. 4. When compared to a control, protein expression of NLRP3, caspase-1 and GSDMD increased in neurones (*p* < 0.001), whereas expressions of caspase-1 and GSDMD increased in astrocytes and microglia (*p* < 0.001; Fig. 2a–f) in the ipsilateral cortex of MCAO mice. Treatment with Li^+^ effectively inhibited increased expressions of NLRP3, caspase-1 and GSDMD as compared with MCAO group (*p* < 0.001; Fig. 2a–f). Protein levels of pro-caspase-1 and ASC were not significantly changed in MCAO mice with or without Li^+^ administration (Fig. 2f, g).

### Signalling pathways involved in Li^+^ neuroprotection

**Fig. 3:**
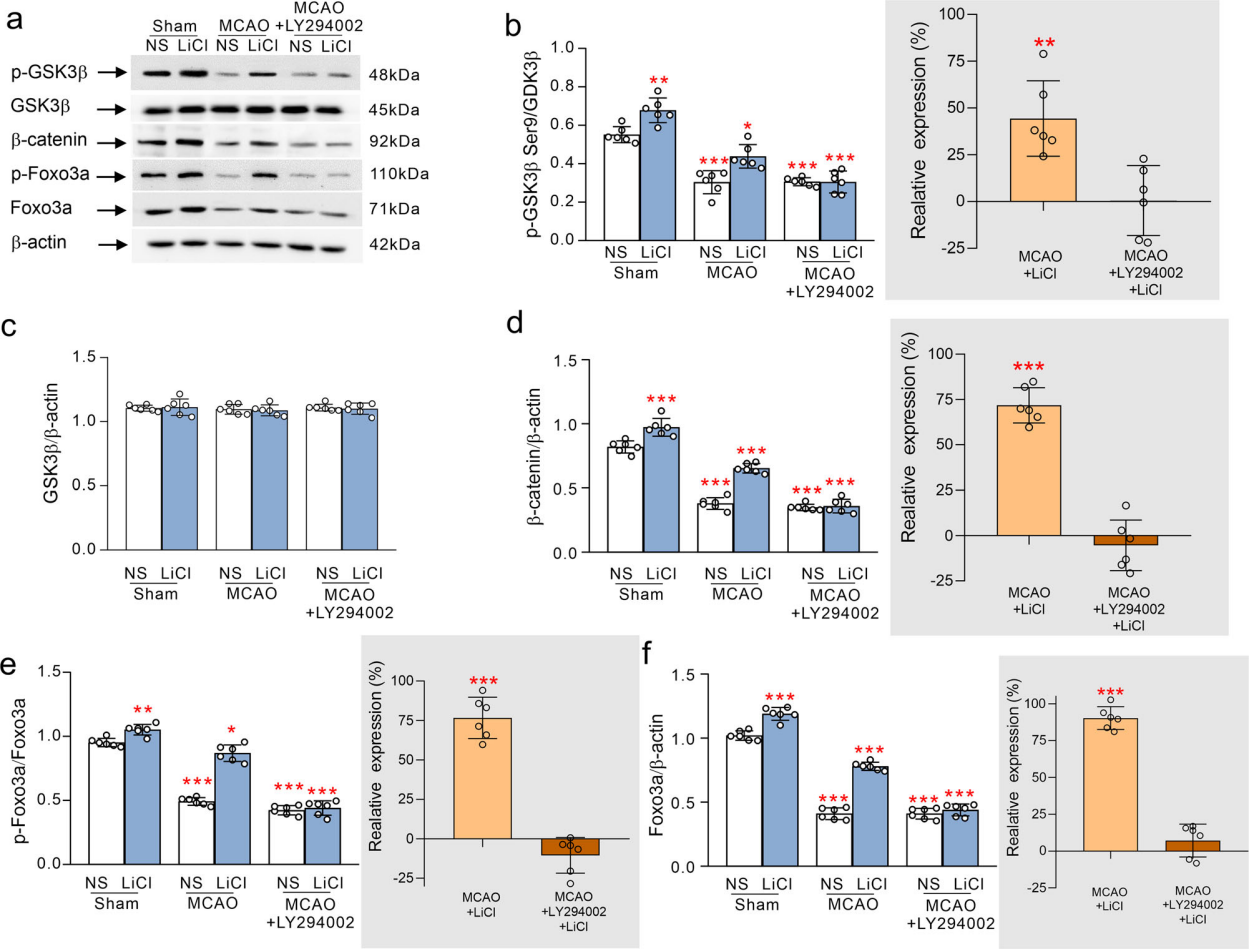
The regulations of Li^+^ in the cytoplasmic levels of GSK3β, β-catenin and FoxO3a by stimulating AKT following ischaemia-reperfusion. **a** Representative protein bands for p-GSK3β, GSK3β, β-catenin, p-FoxO3a, FoxO3a and β-actin (the house-keeping protein) in cytoplasm. **b**–**f** Relative ratio of p-GSK3β and GSK3β is shown in **b**, the relative ratio of p-FoxO3a and FoxO3a is shown in **e**, and the normalised intensities of GSK3β, β-catenin, FoxO3a by β-actin are shown in **c**, **d** and **f**, inserts on the right are the relative comparison with the respective MCAO groups. The protein levels were calculated and plotted as mean ± SD. *N* = 6 per group. Two-way ANOVA for comparisons including more than two groups; unpaired two-tailed *t*-test for two group comparisons. *p < 0.05, **p < 0.01, ***p < 0.001.

**Fig. 4:**
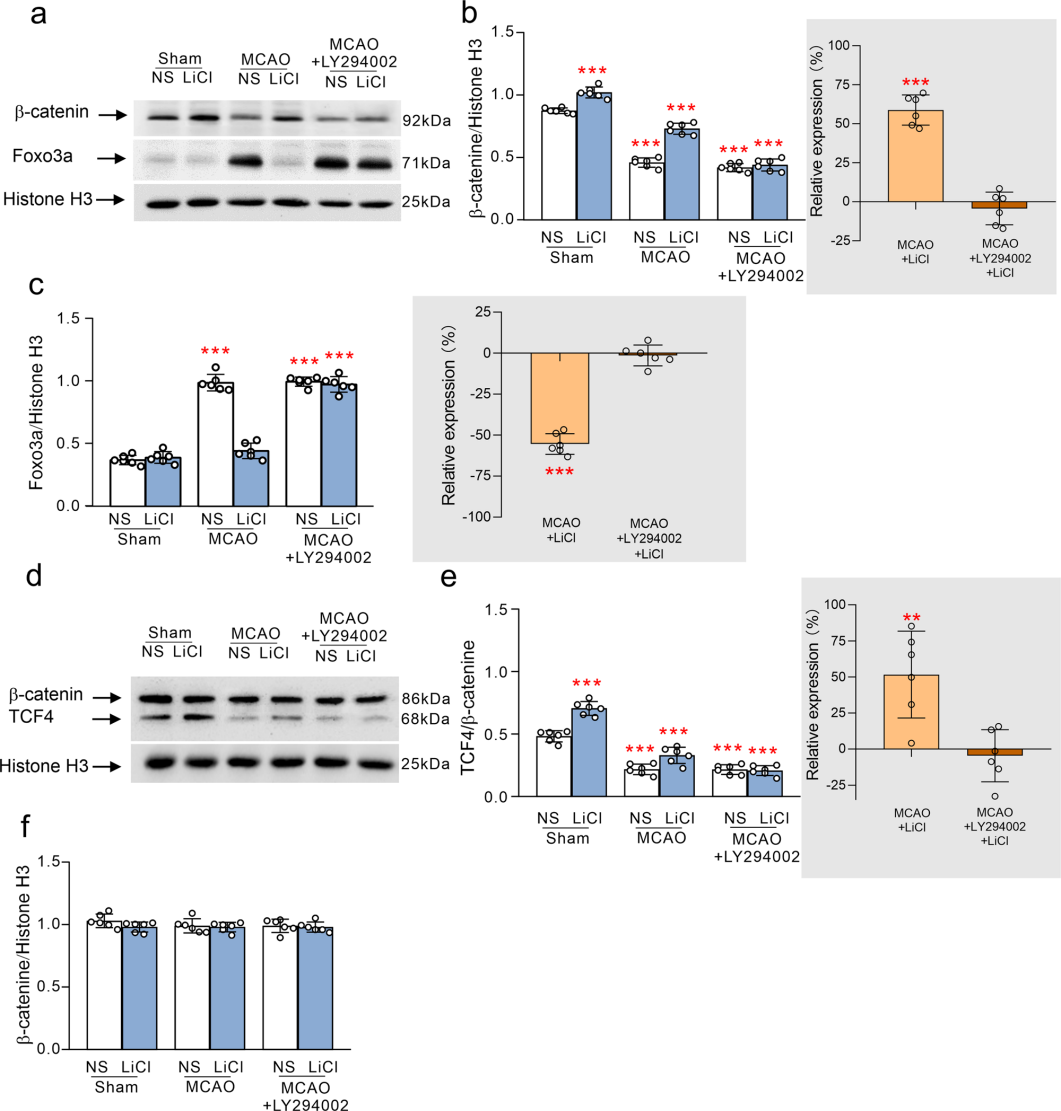
The effects of Li^+^ in the nuclear levels of β-catenin, FoxO3a and TCF4 by stimulating AKT following ischaemia-reperfusion. **a** The representative protein bands of β-catenin, FoxO3a and Histone H3 (the house-keeping protein) in nucleus. The normalised intensities of β-catenin and FoxO3a by Histone H3 were shown in **b** and **c**, inserts on the right are the relative comparison with the respective MCAO groups. **d** The representative protein bands of β-catenin, TCF4 and Histone-H3 (the house-keeping protein) in nucleus. The binding ratio of TCF4 with β-catenin is shown in **e**, and the normalised intensities of Histone H3 by β-actin are shown in **f**, the right insert shows comparison with the respective MCAO groups. The protein levels were calculated and plotted as mean ± SD. *N* = 6 per group. Two-way ANOVA for comparisons including more than two groups; unpaired two-tailed *t*-test for two group comparisons. *p < 0.05, **p < 0.01, ***p < 0.001.

As compared with control, exposure to MCAO decreased the phosphorylation of GSK3β at Ser9 in the extract of the cytoplasm of ipsilateral cortex (*p* < 0.001). Τreatment with Li^+^ increased the phosphorylation of GSK3β Ser9 as compared with MCAO (*p* < 0.01), whereas selective AKT inhibitor (LY294002 at 12.5 mg/kg) abolished action of Li^+^ on the phosphorylation of GSK3β Ser9 (Fig. 3a, b). When compared with control, the expression of GSK3β did not change significantly in the MCAO mice with or without Li^+^ treatment (Fig. 3a, c). Treatment with Li^+^ activates AKT phosphorylation, thus inhibiting GSK3β activity, which, in turn, alleviates the over-activation of GSK3β following ischaemia-reperfusion (Fig. 3a, b). Of note, activation of GSK3β phosphorylates β-catenin resulting in proteosomal degradation^37^, while stabilised β-catenin promptly translocates into the nuclei^38^. We found a significant decrease in cytoplasmic β-catenin in cytoplasm following ischaemia-reperfusion (*p* < 0.001; Fig. 3a, d), likewise, β-catenin was reduced in the nuclear fraction (*p* < 0.001; Fig. 4a, b). When compared with MCAO group, Li^+^ increased β-catenin in cytoplasm and in nucleus, while administration of 12.5 mg/kg LY294002 inhibited Li^+^-induced elevation of β-catenin in cytoplasm and in nucleus (*p* < 0.001; Figs. 3d and 4b). It is known that AKT can phosphorylate transcription factor forkhead box, class O3a (FoxO3a), which results in the redistribution of FoxO3a from the nucleus to the cytoplasm^39^. As compared with control group, exposure to ischaemia-reperfusion decreased the level of FoxO3a in the cytoplasm (*p* < 0.001; Fig. 3a, e, f), but increased its presence in the nucleus (*p* < 0.001; Fig. 4a, c). Phosphorylation of FoxO3a was also decreased in the cytoplasm extracted from the cortex of MCAO treated mice as compared with sham plus NS group (*p* < 0.001; Fig. 3a, f). Treatment with Li^+^ in control group also increased cytoplasmic FoxO3a and p-FoxO3a, while FoxO3a in the nucleus was decreased (Figs. 3e, f and 4c). Treatment with PI3 kinase/AKT inhibitor LY294002 completely eliminated effects of Li^+^ on redistribution of FoxO3a (Figs. 3e, f and 4c). Ischaemia-reperfusion also decreased the co-immunoprecipitation of transcription factor TCF4 with β-catenin (β-catenin and TCF4 complex is known to regulate several downstream genes^40^ as compared with sham group (*p* < 0.001; Fig. 4d, e), whereas Li^+^ enhanced linkage of TCF4 with β-catenin (*p* < 0.001; Fig. 4d, e), again this effect was abolished by LY294002 (Fig. 4d, f).

**Fig. 5:**
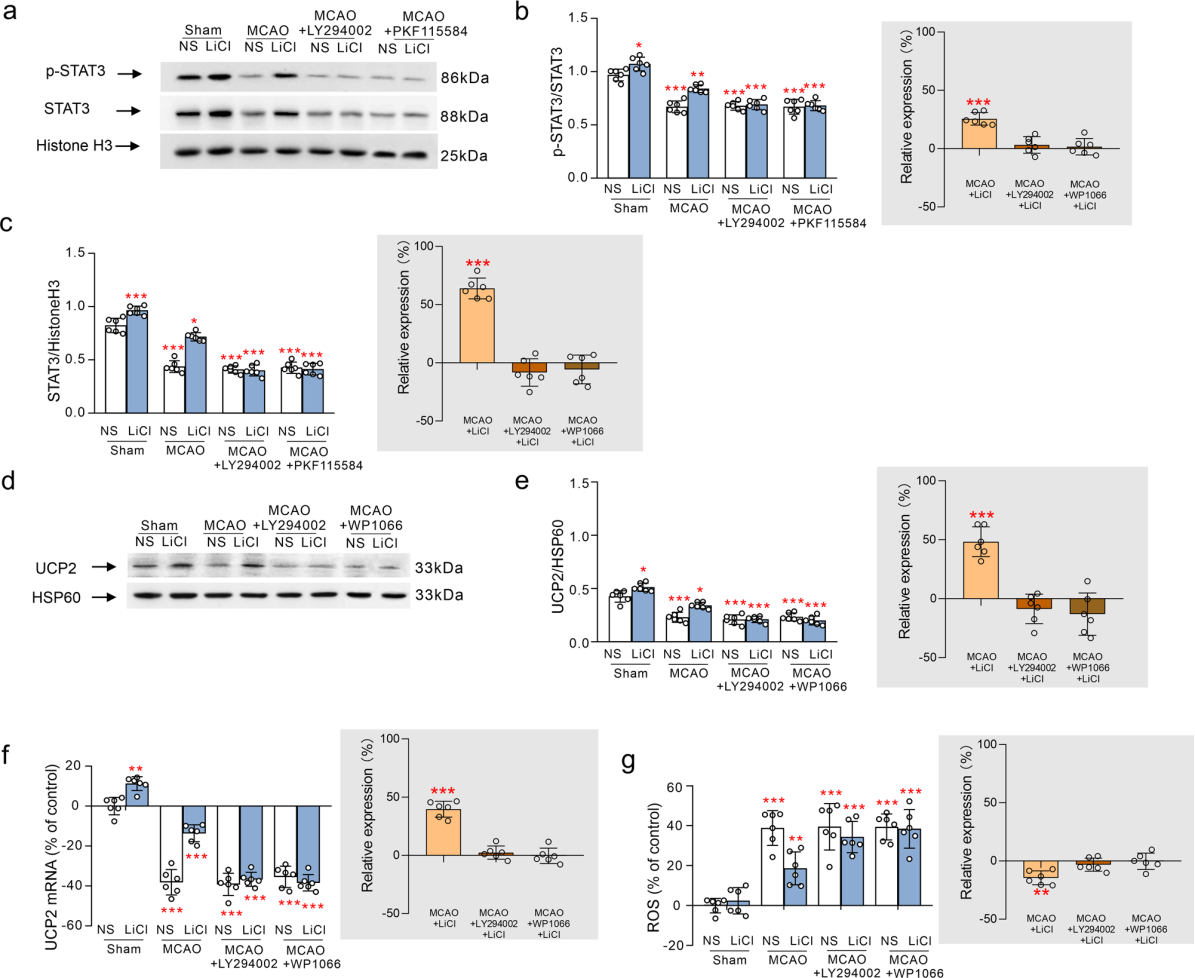
The regulations of Li^+^ in the nuclear levels of STAT3 and the mitochondrial levels of UCP2 and ROS following ischaemia-reperfusion. **a** The representative protein bands of p-STAT3, STAT3 and Histone-H3 (the house-keeping protein) in the nucleus. The phosphorylation ratio of p-STAT3 and STAT3 is shown in **b**, and the normalised intensities of STAT3 by Histone H3 were shown in **c**, the right inserts were the relative comparison with the respective MCAO-NS groups. **d** The representative protein bands of UCP2 and HSP60 (the house-keeping protein) in mitochondrion. The intensity ratio of UCP2 and HSP60 was shown in **e**, and the right insert was the relative comparison with the respective MCAO-NS groups. **f** The relative mRNA expressional ratio of UCP2 normalised by GAPDH (the house-keeping gene). **g** The ROS level normalised to control group. The protein levels were calculated and plotted as mean ± SD. *N* = 6 per group. Two-way ANOVA for comparisons including more than two groups; unpaired two-tailed *t*-test for two group comparisons. *p < 0.05, **p < 0.01, ***p < 0.001.

Protein expression of STAT3 as well as ratio of p-STAT3/STAT3 were decreased by ischaemia-reperfusion (*p* < 0.001; Fig. 5a–c). Administration of Li^+^ increased the expression and phosphorylation of STAT3 in both sham (*p* < 0.05) or MCAO group (*p* < 0.001; Fig. 5a–c). Enhanced expression and phosphorylation of STAT3 induced by Li^+^ was abolished by LY294002 and by the coupling blocker of the complex of β-catenin and TCF4, PKF115584 (Fig. 5a–c). Ischaemia-reperfusion significantly reduced protein and mRNA expression of uncoupling protein 2 (UCP2, mitochondrial cationic carrier protein). Treatment with Li^+^ increased UCP2 expression at protein and mRNA level in both control and MCAO group (*p* < 0.001; Fig. 5d–f). These effects of Li^+^ were suppressed by LY294002 and a selective inhibitor of STAT3, WP1066, (Fig. 5d–f). Finally, MCAO-elevated ROS in the extracted mitochondria from the ischaemic ipsilateral cortex were also decreased by Li^+^, and this effect was antagonised by LY294002 and WP1066 (Fig. 5g).

**Fig. 6:**
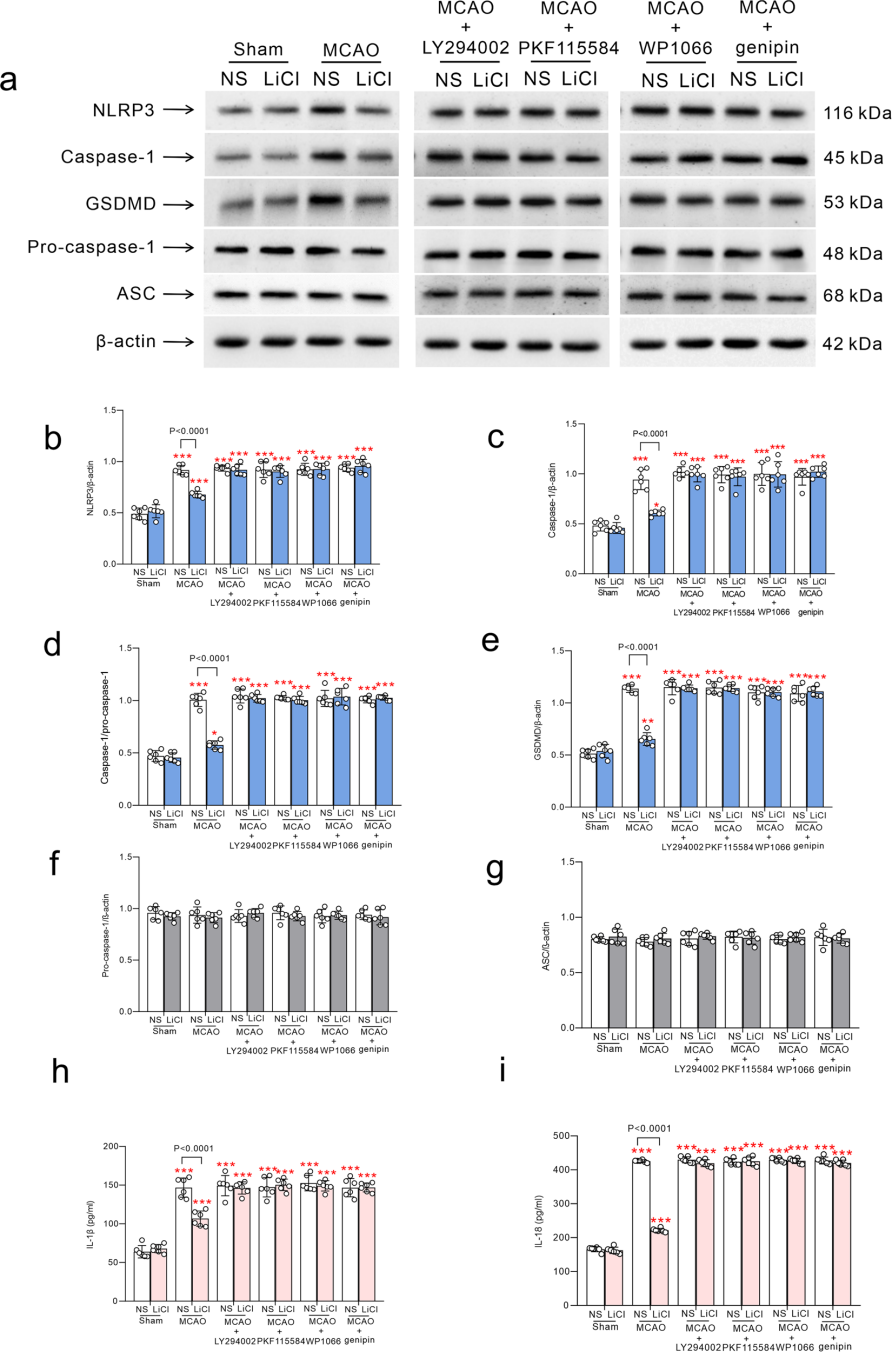
Mechanism of Li^+^ action on NLRP3 and GSDMD following ischaemia-reperfusion. **a** Representative protein bands of NRLP3, caspase-1, GSDMD, pro-caspase-1, ASC and β-actin (the house-keeping protein) in neurones. The normalised intensities of NLRP3, caspase-1, GSDMD, pro-caspase-1 and ASC by β-actin and the ratio of caspase-1 and pro-caspase-1 were shown in **b**–**g**, respectively. The levels of IL-1β and IL-18 measured by ELISA were shown in **h** and **i**. The columns were calculated and plotted as mean ± SD. *N* = 6 per group. Two-way ANOVA for comparisons including more than two groups; unpaired two-tailed *t*-test for two group comparisons. *p < 0.05, **p < 0.01, ***p < 0.001.

Treatment with Li^+^ alleviated MCAO-induced increase in protein expressions of NLRP3, caspase-1 and GSDMD; these effects were antagonised by LY294002, PKF115584, WP1066 and UCP2 inhibitor genipin (Fig. 6a–e). Protein expressions of pro-caspase-1 and ASC were not significantly modulated by MCAO or the administration of Li^+^ (Fig. 6f, g). In contrast, production of IL-1β and IL-18 by NLRP3 inflammasome after ischaemia-reperfusion was reduced by Li^+^, which was prevented by LY294002 and WP1066 and genipin (Fig. 6h, i).

### The neuroprotective effects of Li^+^ on ischaemic stroke

**Fig. 7:**
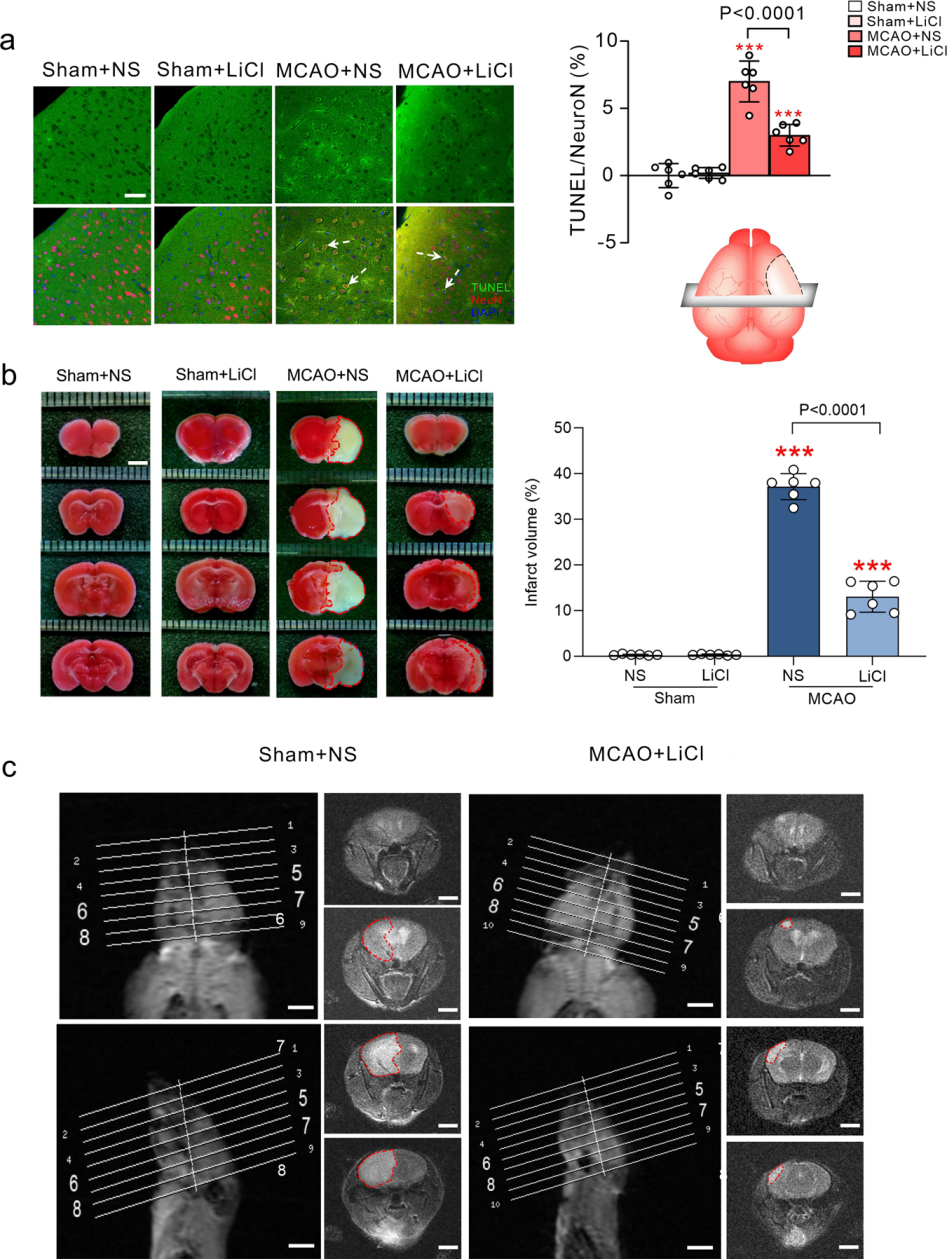
Reduction of neuronal apoptosis and ischaemic volume by Li^+^. **a** TUNEL co-staining with neuronal marker NeuN and nucleus marker DAPI, and the relative ratio of Tunel-positive cells in NeuN-positive cells. Scar = 50 μm. **b** TTC staining in brain slices, and the percentage of infarct volume. Scar = 3 mm. **c** The representative images show ischaemic regions on T2wt MRI. The T2wt MRI images of MCAO built mice treated with NS or LiCl. Scar = 5 mm. The percentage of neuronal apoptosis and ischaemic volume were calculated and plotted as mean ± SD. *N* = 6 per group. One-way ANOVA for comparisons including more than two groups; unpaired two-tailed *t*-test for two group comparisons. *p < 0.05, **p < 0.01, ***p < 0.001.

Neuronal apoptosis in the ischaemic penumbra in MCAO mice was significantly alleviated by Li^+^ (*p* < 0.001; Fig. 7a). Likewise, the ischaemic volume (assessed by staining with 1% 2,3,5-triphenyltetrazolium chloride, TTC) was significantly reduced by Li^+^ (*p* < 0.001; Fig. 7b). Similar decrease in ischaemic region in the presence of Li^+^ was observed in T2-weighted magnetic resonance (MRI) images (Fig. 7c). Positive impact of Li^+^ on the neuronal apoptosis and ischaemic volume triggered by MCAO, are negated by the pre-treatment with the antagonists of LY294002, PKF115584, WP1066 and genipin (Supplementary Figs. 6 and 7).

**Fig. 8:**
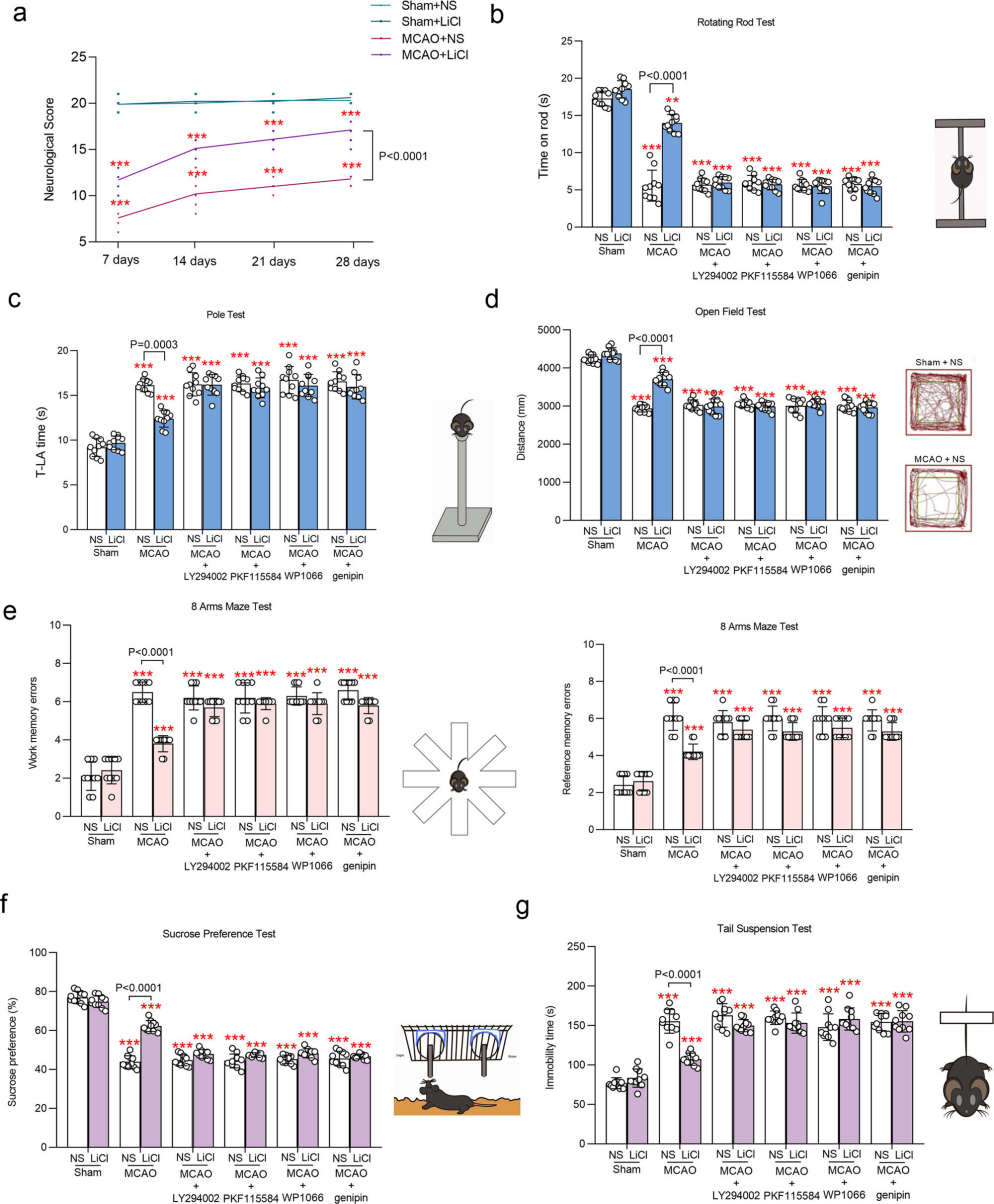
Behavioural effects of LiCl. **a** The neurological scores measured at 7, 14, 21 and 28 days. **b** The staying time on rod measured by rotating-rod test. **c** T-LA time measured by Pole test. **d** The move distance was recorded in open-field test. **e** The work memory errors and reference memory errors were calculated in 8-arms maze test. **f** The sucrose preference percentage were measured by sucrose preference test. **g** The immobility time were recorded in tail-suspension test. The neurological score and the evaluated behavioural indicators were calculated and plotted as mean ± SD. *N* = 10 per group. Two-way ANOVA for comparisons including more than two groups; unpaired two-tailed *t*-test for two group comparisons. *p < 0.05, **p < 0.01, ***p < 0.001.

Treatment with Li^+^ improved the 4-week dynamic of neurological scores of MCAO mice (Fig. 8a). In particular, Li^+^ significantly improved motor activities of MCAO mice as revealed by rotating rod and pole tests; these improvements were antagonised by LY294002, PKF115584, WP1066 and genipin (Fig. 8b, c). Similarly, Li^+^ improved MCAO-affected outcomes of open-field test *t* (Fig. 8d), and decreased the number of work and reference memory errors recorded in 8-arms maze test (Fig. 8e). Finally, Li^+^ alleviated depressive-like behaviours induced by MCAO, as demonstrated by sucrose preference and tail-suspension test (Fig. 8f); Li^+^ also reduced the immobility time elevated in MCAO mice (Fig. 8g). All these effects of Li^+^ were abolished by LY294002, PKF115584, WP1066 and genipin (Fig. 8b–g).

## Discussion

**Fig. 9:**
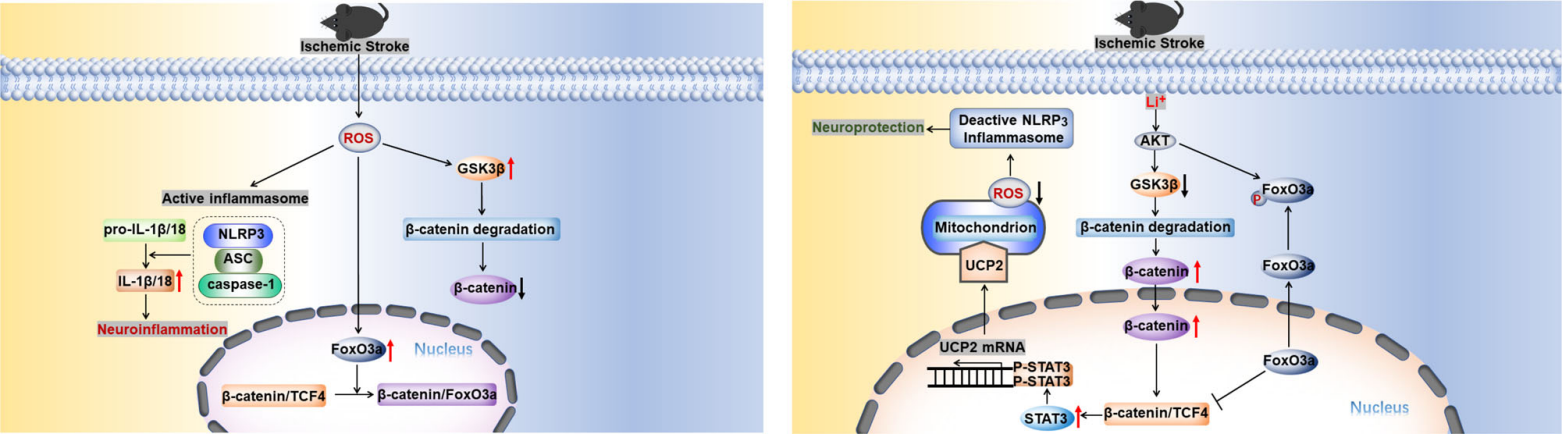
Molecular pathways of Li^+^ neuroprotection following ischaemia-reperfusion. Under the pathological condition of ischaemic stroke, LiCl inhibited the activation of GSK3β by phosphorylating AKT, then the degradation of β-catenin was decreased, the increased β-catenin in cytoplasm more easily translocated into nucleus. As well as, LiCl increased the phosphorylation of FoxO3a by activating AKT, which promoted the more FoxO3a transportation from nucleus into cytoplasm, and the reduced FoxO3a in nucleus lacked the competition with TCF4, the increased complex level of β-catenin and TCF4 stimulated the expression and the phosphorylation of STAT3, which further induce the mRNA and protein expression of UCP2. In mitochondrion, the increased UCP2 suppressed the production of ROS and resulted in the deactivation of NLRP3 inflammasome stimulated by I/R injuries, in order to play neuroprotective role of lithium.

In this study, we dissected molecular mechanisms of Li^+^ neuroprotection. We discovered that Li^+^ effectively suppresses activation of NLRP3 inflammasome in the ischaemic cortex and improves impaired behaviours. At a molecular level, Li^+^ inhibits activation of GSK3β by stimulating AKT, inactivated GSK3β subsequently decreases degradation of cytoplasmic β-catenin, which is transferred into the nucleus to form β-catenin/TCF4 complexes. At the same time AKT, stimulated by Li^+^, increases phosphorylation of cytoplasmic FoxO3a, which results in FoxO3a exit from the nucleus. Nuclear FoxO3a competes with binding sites of β-catenin with TCF4, and therefore the decrease of nuclear FoxO3a induced by Li^+^, further enhances formation of β-catenin/TCF4 complexes. These complexes phosphorylate STAT3, which promotes expression of UCP2. The up-regulation of UCP2 decreases production of ROS in mitochondria, which counteracts the activation of NLRP3 inflammasome induced by ischaemia and reperfusion (Fig. 9).

Our study suggests that Li^+^ acts through signalling pathways responsible for neuroinflammation and pyroptosis, in particular interfering with ischaemia- reperfusion-induced activation of NLRP3 inflammasome. Activation of NLRP3 inflammasome was detected only in neurones, whereas increase in pyroptosis executor gasdermin D has been identified in neurones, astrocytes and microglia of ischaemic cortex. Administration of Li^+^ effectively suppressed activation of NLRP3 inflammasome and neuronal apoptosis. However, increase in NLRP3 protein expression induced by ischaemia and reperfusion in the cortex only occurred in neurones and not in astrocytes or microglia. Our data highlight that activation patterns of NLRP3 inflammasome induced by ischaemia and reperfusion are different between neurones and glia. Similarly, inhibition of inflammasome complex NLRP1 was also reported to alleviate the neuroinflammation after thromboembolic stroke in mice^41^. Increased activation of NLRP3 inflammasome may even indicate detrimental outcomes in patients suffered ischaemic stroke^42,43^. After ischaemia-reperfusion, activation of caspase-1 recruitment of GSDMD was NLRP3-independent in astrocytes and in microglia. Similarly, in macrophages and neurotrophils, NLR-family CARD-containing protein 4 (NLRC4) is reported to activate caspase-1 and trigger pyroptosis^44^. Meanwhile, after injuries, Neuronal apoptosis induced by ischaemia-reperfusion is associated with the neuroinflammation^45^, while oxidative stress induced by the injuries could stimulate the neuroinflammatory cascades, including activations of NLRP3 inflammasome and secretion of inflammatory cytokines, IL-1β, IL-18, TNF-α and IL-6^46^. Pro-inflammatory cytokines may further exacerbate neuronal apoptosis resulting in behavioural deficits^47,48^.

FoxO3a is a transcriptional target for mood disorder treatments, while FoxO3a-deficient mice present antidepressant-like behaviours^49^. In cultured embryonic kidney (HEK 293) and SH-SY5Y neuroblastoma cells, the antidepressive action of Li^+^ involves reduced FoxO3a transcription and decreased protein levels both in cytosol and nucleus^50^. We observed positive effects of Li^+^ not only on cognition and motor abilities, but also on despair-like behaviours induced by ischaemia-reperfusion. In rats exposed to chronic mild stress (CMS), depressive-like behaviours are associated with reactive microgliosis with an increase in NF-κB, TNF-α and IL-1β in hippocampus. Chronic treatments with LiCl (50 mg/kg/day; i.p.) for 4 weeks also increase the accumulation of β-catenin by inhibiting GSK-3β and alleviate the microglial inflammation induced by CMS^51^. Chromic stress also reduced expression of aquaporin-4 (AQP4), while administration of Li^+^ at the serum levels around 0.61 mmol/L restored AQP4^52^. Furthermore, the post ischaemia treatment with lithium could alleviate BBB injury after intracerebral haemorrhage in rats by regulating AKT/GSK-3β^53^. In this study, we further demonstrate that Li^+^ acting through AKT pathway enhances the phosphorylation of FoxO3a, which promotes the translocation of FoxO3a from nucleus into cytoplasm, thus suppressing competition of FoxO3a with TCF4 for the binding points of β-catenin in nucleus.

It is known that Li^+^ restores compromised blood-brain barrier (BBB) by up-regulating endothelial Wnt/β-catenin signalling^54^. Increase in β-catenin/TCF4 complex formation induced by Li^+^ accelerates the expression and phosphorylation of STAT3 in the nucleus (Fig. 5). Furthermore, WP1160, a STAT3 agonist, decreased NLRP3 expression by reducing the downstream STAT3 activation in SH-SY5Y neuroblastoma^55^. Our previous research also demonstrates that the increased activation of STAT3 by fluoxetine and leptin restores the sleep deprivation-induced depressive-like behaviours by inhibiting an activation of NLRP3 inflammasome in astrocytes^56,57^. In the present study we also show that Li^+^ inhibits activation of NLRP3 inflammasome and improves motor behaviour, cognition and depression by stimulating STAT3 (Figs. 6 and 8). This inhibition of STAT3 results in the up-regulation of expression of UCP2 (Fig. 5). In UCP2-knockout mice, activation of NLRP3 inflammasome is enhanced by increased ROS production, which exacerbates detrimental effects of ischaemia and reperfusion^58^.

The neuroprotection of Li^+^ is also linked to an increase in BDNF production, activation MAPK/ERK_1/2_ pathway, or elevation of the miRNA-124 expression^59–61^. All in all our in depth analysis of Li^+^-imposed neuroprotection in the context of ischaemia-reperfusion provide the basis for clinical application of Li^+^ in ischaemia and stroke.

## Methods

### Materials

Primary antibody of ASC (sc-365611), caspase-1(sc-56036), β-catenin (sc-59737) and p-STAT3 (sc-81523) were purchase from Santa Cruz Biotechnology (Santa Cruz, CA, USA). Primary antibody of NLPR3 (ab214185), pro-caspase-1 (ab179515), GFAP (ab48050), Iba-1 antibody used for FACS (ab178846) and secondary antibody Alexa Fluor 555 goat anti-rabbit (ab150074) were purchased from Abcam (Cambridge, MA, USA). Primary antibody of Foxo3a (720128), p-GSK3β Ser9 (MA5-14873), UCP2 (PA5-80203), NeuN (PA5-78693) and Alexa Fluor-conjugated 488 secondary antibody used for FACS (A-11001) were purchased from Thermo Fisher Scientific (Waltham, MA USA). Primary antibody of GSK3β (22104-1-AP), TCF4 (22337-1-AP) and STAT3 (10253-2-AP) were purchased from Proteintech (Wuhan, Hubei, China). Primary antibody of β-actin (E021020), Histone H3 (E021130), HSP60 (A200659) and secondary antibody HRP-labelled Goat anti-Mouse (E030110) and HRP-labelled Goat anti-Rabbit (E030120) were purchased from Earthox (Millbrae, CA, USA). LY294002 was from PeproTech (1543664, Rocky Hill, NJ, USA). PKF115584 was from Novartis (Basel, Switzerland), WP1066 (S2796) and geninpin (S2412) was from Selleck Chemicals (Houston, Texas, USA). Iba-1 antibody used for immunofluorescence (019-19741) was purchased from Wako Chemicals (USA) Chemicals for the preparation of cell-sorting medium, p-Foxo3a antibody (PA5-37578) and Alexa Fluor-conjugated 488/555 secondary antibody (A21202, A31570) were from Gibco Life Technology Invitrogen (Grand Island, NY, USA). 2,3,5-triphenyltetrazolium chloride (T8877) was from Sigma-Aldrich (St. Louis, Missouri, USA). TUNEL apoptosis detection kit (11772457001) was purchased from Roche-Sigma (USA). The ROS assay kit (BB-470532) was purchased from BestBio (Shanghai, China). The quantitative PCR (RR820A and RR047A) was purchased from TaKaRa Bio (Kusatsu, Japanese). The enzyme-linked immunosorbent Assay (ELISA) kit for IL-1β (88-7013) and IL-18 (88-50618) were purchased from Thermo Fisher Scientific (Waltham, MA USA). The mitochondria extraction kit (BB-470532) was purchased from BestBio (Shanghai, China).

### Animals

The male wild-type C57BL/6 mice (#000664), FVB/N-Tg(GFAP-eGFP)14Mes/J (#003257) and B6.Cg-Tg(Thy1-YFP)HJrs/J (#003782) transgenic mice were all purchased from the Jackson Laboratory (Bar Harbor, ME, USA). The animals were raised in standard housing conditions (22 ± 1 °C; light/dark cycle of 12/12 h), with water and food available *ad libitum*. All experiments were performed in accordance with the US National Institutes of Health Guide for the Care and Use of Laboratory Animals (NIH Publication No. 8023) and its 1978 revision, and all experimental protocols were approved by the Institutional Animal Care and Use Committee of China Medical University, No. [2020]102.

### Middle cerebral-artery occlusion (MCAO)

MCAO was used to model ischaemic stroke in mice. The male C57BL/6 mice, FVB/N-Tg(GFAP-eGFP)14Mes/J mice or B6.Cg-Tg(Thy1-YFP)HJrs/J transgenic mice (aged 10-12 weeks; weight of 25–35 g) were anesthetised with ketamine (80 mg/kg, i.p.) and xylazine (10 mg/kg, i.p.) and placed on their backs to expose the neck area. A 1.5 cm incision was made in the middle of neck. The right common artery (CCA) was exposed. The external arteries (ECA) and internal carotid arteries (ICA) were isolated from CCA. The distal ECA was tied off and the CCA was clipped by a bulldog clamp. The ECA was opened by arteriotomy and a 5-0 nylon monofilament (181022, LingQiao, Ningbo, Zhejiang, China) was inserted and gently advanced upwards to the ICA. The insertion was stopped when resistance was felt. After 60 min of occlusion, the monofilament was withdrawn to allow for reperfusion. The mice were dislocated executed to collect tissues, after building MCAO models with reperfusion 5 or 28 days. In sham groups, the carotid arteries were exposed and the skin was sutured. Body temperature of mice was maintained and mice were individually housed after full recovery from anaesthesia^62^.

### Experimental design and drug administration

The wild-type or transgenic mice were randomly segregated into sham plus normal solution (NS) group (control), sham plus lithium chloride group (LiCl), MCAO plus NS group (MCAO) and MCAO plus LiCl group. 30 min before the surgery of MCAO, some mice in MCAO groups were randomly chosen to be intraperitoneal injected with 12.5 mg/kg/day LY294002 (AKT inhibitor), 0.2 mg/kg/day PKF115584 (antagonist of β-catenin/TCF4 complex), 30 mg/kg/day WP1066 (STAT3 inhibitor) or 2.5 mg/kg/day geninpin (UCP2 inhibitor). After 30 min, mice with or without injected inhibitors were subjected to MCAO or treated with sham; 30 min after surgery 1 mmol/kg/day LiCl or normal saline (NS) were randomly intraperitoneally injected. After 60 min of ischaemia, the middle cerebral artery was reperfused, The injection of all reagents was repeated once a day for 5 days. Subsequently, 6 mice form each group were sacrificed by cervical dislocation for histology and biochemistry. Remaining mice were used for behavioural tests; detailed experimental design is shown in Supplementary Fig. 1.

### Immunofluorescence (IF)

To identify the levels of NLRP3, caspase-1, GSDMD in three nerve cells (neurones, astrocytes and microglia) in ischaemic penumbra area of cerebral cortex. The anaesthetised mice were trans-myocardially perfused with 4% paraformaldehyde (PFA) for 15 min. The collected brains were fixed by immersion in 4% paraformaldehyde and cut into 60 μm slices. The slices were permeabilised by incubation for 1 h with donkey serum. Primary antibodies against NLRP3, caspase-1 or GSDMD were used at 1:200 dilution, against glial fibrillary acidic protein (GFAP), NeuN or Iba1 was used at 1:200 dilution. The nuclei were stained with marker 4’, 6’-diamidino-2-phenylindole (DAPI) at 1:1000 dilution. The incubation with the primary antibodies were overnight at 4 °C and then donkey anti-mouse or anti-rabbit Alexa Fluor 488/555 conjugated secondary antibodies were incubated for 2 h at room temperature. Images were captured using a confocal scanning microscope (DMi8, Leica, Wetzlar, Germany)^63^. The immunofluorescence intensity of NLRP3, caspase-1 and GSDMD was co-localised with the cell-specific markers (NeuN, GFAP and Iba1). Background intensities per image were measured in cell-free parenchyma in the same field of view and subtracted from the total immunofluorescence intensities. The intensity of NLRP3, caspase-1 or GSDMD immunofluorescence from the different groups was normalised to the intensity of the control (sham + NS) group.

### Co-immunoprecipitation (Co-IP)

We used technologies of co-immunoprecipitation and subsequent western blotting to check the conjunction level between TCF4 and β-catenin. For immunoprecipitation of TCF4, lysates of mice cerebral cortex (500 μg) were incubated with 20 μg of β-catenin antibody for overnight at 4 °C, meanwhile, 20 μg of the mouse normal IgG was used as negative control (Supplementary Fig. 5). Then, 200 μl of washed protein G-agarose bead slurry was added, and the mixture was incubated for another 2 h at 4 °C. The agarose beads were washed three times with cold phosphate buffer solution (PBS) and collected by pulsed centrifugation (5 s in a microcentrifuge at 14,000 × *g*), the supernatant was drained off, and the beads were boiled for 5 min. Thereafter, the supernatant was collected by pulsed centrifugation. Then, the protein content was determined by the Bradford method^64^ using bovine serum albumin (BSA) as the standard again. 100 μg immunoprecipitates were subjected to 10% sodium dodecyl sulfate (SDS)-polyacrylamide gel electrophoresis (PAGE) and followed the next measurements of western blotting^63^.

### Western blotting

Protein expressions of NLRP3, pro-caspase-1, caspase-1, GSDMD, p-GSK3β Ser9, GSK3β, β-catenin, TCF4, p-STAT3, STAT3, UCP2, HSP60, β-actin and Histone H3 were analysed with Western blot performed 5 days after MCAO or sham surgeries. The samples of ischaemic ipsilateral cortex containing 100 μg of protein were added 10% SDS-polyacrylamide gel electrophoresis. After electrophoretic separation the gels were transferred to polyvinylidene fluoride (PVDF) membranes, the membranes were blocked by 5 % skimmed milk powder for 1 h, and incubated overnight with the primary antibodies, specific to: NLRP3 at 1:1000 dilution, pro-caspase-1 at 1:1000 dilution, caspase-1 at 1:1000 dilution, GSDMD at 1:1000 dilution, p-GSK3β Ser9 at 1:1000 dilution, GSK3β at 1:3000 dilution, ASC at 1:1000 dilution, β-catenin at 1:1000 dilution, p-Foxo3a at 1:1000 dilution, Foxo3a at 1:1000 dilution, TCF4 at 1:1000 dilution, STAT3 at 1:3000 dilution, p-STAT3 at 1:1000 dilution, UCP2 at 1:1000 dilution, HSP60 at 1:3000 dilution, β-actin at 1:3000 dilution and Histone H3 at 1:3000 dilution. After washing, specific binding was detected by horseradish peroxidase-conjugated secondary antibodies. Staining was visualised by electrochemiluminescence (ECL) detection reagents and were analysed with an Electrophoresis Gel Imaging Analysis System (MF-ChemiBIS 3.2, DNR Bio-Imaging Systems, Israel). Band density was measured with Window AlphaEaseTM FC 32-bit software^65^.

### Real-time PCR

To measure the mRNA of UCP2, the RNA of cerebral cortex was extracted by Trizol reagent (Invitrogen, Carlsbad, CA, USA). Total RNA was reverse transcribed to cDNA by using a reverse transcription reagent kit (Takara, Otsu, Shiga, Japan) in a Robo-cycler thermocycler. Real-time PCR was performed with the LightCycler 480 SYBR Green I Master kit and the products were detected using an a LightCyler 480 instrument. The sequences of Real-time PCR primers used for mRNA quantification were as follows: 5′-TAGTGCGCACCGCAGCC-3′ and 5′-AGCTCATCTG-GCGCTGCAG-3′ for mouse UCP2 and 5′-TGGTGCCAAAAGGGTCATCTCC-3′ and 5′-GCCAGCCCCAGCATCAAAGGTG-3′ for mouse GAPDH. Relative genomic expression was calculated by the 2^−ΔΔCt^ method^66,67^.

### The proteins extraction of cytoplasm and nucleus

The cytoplasm or nuclear proteins were extracted by using the protein extraction kit, according to the manufacturer’s protocol. The collected cortex was cut into small pieces and placed in new pre-cooling microcentrifuge tubes. Then, 200 μl mixture solution of cytoplasmic extraction reagent A and B was added, and the tissues were grinded into homogenate. All sample tubes were placed in an ice-bath and rest for 10 min. 10 μl cytoplasmic extraction reagent B was added again and the sample was vortexed and centrifugated at 15,000 × *g*, 4 °C for 5 min. After centrifugation, the cytoplasmic extract in supernatant was immediately transferred to a new pre-cooled tube, and the sediment was suspended with 50 μl nuclear extraction reagent. The sediment was vortexed for 30 seconds. Then, the sample tubes were put back into the ice-bath and vortexed for 30 seconds every 2 min, keeping 30 min. Centrifuging the sample tubes at 15,000 × *g*, 4 °C for 10 min, the supernatant was obtained as nuclear extract.

### Mitochondria isolation

Mitochondria isolation and fractionation was conducted by using the mitochondria extraction kit, according to the manufacturer’s protocol. Fresh cerebral cortex separated from mice brain were homogenised with a glass homogeniser in cold lysis buffer. Then, the homogenates were centrifuged at 800 × *g*, 4 °C for 5 min. After centrifugation, the supernatant was slowly transferred to a new pre-cooled tube, which pre-added with 0.5 ml extraction reagent A. To centrifuge the sample tubes at 15,000 × g, 4 °C for 10 min, the supernatant was obtained as cytoplasm extract. The sediment was suspended with 200 μl wash buffer, then centrifuged at 15,000 × *g*, 4 °C for 10 min again. To dispose the supernatant, the sediment was obtained as mitochondria and resuspended with 100 μl storage buffer and kept under −80 °C.

### ROS detection

To assay the ROS level, a commercial ROS assay kit was used and operated as the protocols. To conduct the experiment, cerebral cortex of mice was homogenised with 1 ml buffer reagent A by using a glass homogeniser. The homogenates were centrifuged at 15,000 × *g*, 4 °C for 10 min. In all, 190 μl supernatant of tissue homogenates and 10 μl fluorescence dye 2′,7′-dichlorodihydrofluorescin diacetate (DCFH-DA) were co-incubated in 96 well plate for 30 min at 37 °C. Finally, the fluorescence intensity of each well was measured using a fluorescence microplate reader (Infinite M200 Pro, Hombrechtikon, Swiss). For fluorescence detection, the excitation wavelength was at 488 nm, and the emission peak was examined at 525 nm.

### ELISA assays

Cytokine levels of IL-1β and IL-18 in cortex were determined in duplicate following ELISA assay instructions provided by the manufacturers. The optical density (OD) of each microwell was measured at 450 nm using a microplate reader. The minimum detection limits for mouse IL-1β and mouse IL-18 were 31.2 pg/ml and 8 pg/ml, respectively.

### Sorting neural cells through fluorescence-activated cell sorter (FACS)

To measure the protein expressions of NLRP3 inflammasome and GSDMD, B6.Cg-Tg(Thy1-YFP)HJrs/J transgenic mice were used for isolating neurones, FVB/N-Tg(GFAPGFP)14Mes/J mice were used for isolating astrocytes, wild-type mice were used for sorting microglia by using Iba-1 primary antibody and the associated Alexa Fluor 488 secondary antibody. A single-cell suspension from the cortex was prepared. The cortices from three mice were pooled for one sample. Wavelengths of 488 nm and 530/30 nm were used for GFP or YFP excitation and emission, respectively. The labelled cells were sorted and collected using the BD FACSAria Cell Sorting System (35 psi sheath pressure, FACSDiva software S/W 2.2.1; BD Biosciences, San Jose´, CA)^56^. As shown in Supplementary Fig. 3, the purity of sorted neuronal, astrocytic or microglial populations has been ascertained by detecting mRNA of cell-specific markers as described previously^36,67–69^.

### TUNEL measurements

The anaesthetised mice were perfused transcardially with 4% paraformaldehyde (PFA) for 15 min. The brain tissue was fixed by immersion in 4% paraformaldehyde and cut into 60 μm slices. Apoptosis in ischaemic penumbra area of cerebral cortex was detected via TUNEL assay kit, as the manufacturer’s protocol. Then, the slices were stained with the neuron marker NeuN at 1:200 dilution followed by incubation with DAPI solution at 1:1000. Images were captured with a confocal scanning microscope (DMi8, Leica, Wetzlar, Germany). The apoptotic cells were calculated as a percentage of the TUNEL-positive cells in the NeuN-positive cells^63^.

### Infarct volume measurement

Mice were executed and each brain was cut into coronal slices (2 mm thick) with a brain-cutting matrix. The slices were stained with 1% 2,3,5-triphenyltetrazolium chloride (TTC) at 37 °C for 10 min in the dark. Normal tissues were stained (red) and infarct tissues were not stained (white). The slices were mounted to a dry black paper and photographed with a video microscopy (Chensheng Optical, Shenzhen, China). By using software ImageJ (ImageJ 1.46r, NIH) to calculate the total area and the ischaemic area, the “A1” is equal to the average of the ischaemic area on the front and back sides of the first brain slice, “B1” is equal to the average of the total area on the front and back sides of the first brain slice, T1 is equal to the thickness of the first brain slice. From these parameters, the ischaemic ratio in the four consecutive brain slices was calculatied using the formula: the percentage of infarct volume = (A1 × T1 + A2 × T2 + A3 × T3 + A4 × T4)/(B1 × T1 + B2 × T2 + B3 × T3 + B4 × T4) × 100%, this percentage of the infarct volume represented as the ischaemic extent.

### Magnetic resonance imaging (MRI)

We performed in vivo MRI experiments on Signa 3.0 T HDxt system (GE Healthcare, Fairfield, Connecticut, USA). Mice were anaesthetised with ketamine (80 mg/kg, i.p.) and xylazine (10 mg/kg, i.p.) and put on an animal scanning bed. T2-weighted (T2 wt.) images were collected by using a fast-spin-echo (FSE) T2-weighted sequence with the following parameters: echo time/repetition time = 85.46/ 4620 ms, field of view = 40 × 40 mm, 10 slices with 1.5 mm slice thickness, in-plane resolution of 512 × 512 pixel, and an imaging time of 12 min^70^.

### Behavioural tests

Mice were subjected to behavioural tests as shown in Supplementary Fig. 1, the test sequences were randomly assigned to avoid interference from different test. Behavioural tests were recorded, stored, and analysed by using the SMART™ tracking software programme (Panlab, SL, Barcelona, Spain). The apparatus was cleaned with 25% ethanol solution after each test.

### Neurological scores (NST)

To assess neurobehavioral deficits of MCAO mice, the 21 points Garcia Score were used. Neurological outcomes of mice were assessed by a blinded observer at 7 days, 14 days, 21 days and 28 days after building MCAO. The 21 points Garcia Score is a sensorimotor assessment system consisting of seven tests with scores of 0-3 for each test. These seven tests included: (i) spontaneous activity, (ii) side stroking, (iii) vibris touch, (iv) limb symmetry, (v) climbing, (vi) lateral turning, and (vii) forelimb walking^71^.

### Rotating-rod test (RRT)

Each mouse was placed on a rotating bar, which was set to a rotation speed of up to 20 rpm during the test. The time spent on the rotating bar was recorded as the latent period. The latency before falling was recorded using a stop-watch, with a maximum of 90 seconds. Time of staying on rotating rod was used to assess motor balance ability^72^.

### Pole test (PT)

Each mouse was paced head-upward on the top of a vertical rough-surfaced pole (diameter 1 cm; height 55 cm). The turn downward from the top of pole (T-turn time) and the descend to the floor (T-LA) time was recorded^73^.

### Open-field test (OFT)

The mice were pre-trained to familiar with the environment. Mice were trained at 26 and 27 days after MCAO operation. At 28 days, formal test was performed. Both in training and testing phase, each mouse was placed in an open-field box (40 cm × 40 cm white floor with black plastic walls 30 cm in height) and allowed to explored for 6 min. Total travel distance was recorded (mm) and used to assess the capacity of mobility^57^.

### 8-arms maze test

The maze was composed of a central platform (32 cm in diameter) from which radiated eight identical arms (50 cm long and 12 cm wide). The entrance to each arm of the maze was controlled by sliding doors that could be controlled manually by an experimenter. Each arm was terminated by a small well in which food rewards were delivered. The food rewards were single feed pallets. Mice were habituated for 2 days. During habituation, single feed pellets were placed in the small well located at the terminal ends of each arm. At the beginning of each habituation trial, mice were placed in the central platform of 8-arm maze and explored for 15 seconds. Then, all doors opened to allow mice to freely explore the maze. Mice that took all baits within a 10 min trial by the last day of habituation were advanced to the training phase. During training, single feed pellets were placed in terminal well of four arms (arms 2, 3, 6 and 7 in clockwise fashion). The baited arms and never-baited arms were kept constant throughout the whole training and testing phase. Working memory error (WME) is defined as re-entering the baiting arm, and reference memory error (RME) is defined as entering the non-baiting arm for the first time. If the WME frequency is zero within five consecutive training trials and the RME is one time or less, the training is considered successful. The mice were trained at 21 days after MCAO operations. Mice with or without MCAO treatments were training for 7 days and performed formal testing at 27 to 28 days after building MCAO model. WME and RME were calculated^74^.

### Sucrose preference test (SPT)

The sucrose preference test was performed 27 days after MCAO. After the deprivation of food and water for 12 h, mice were provided with two pre-weighted bottles, including one bottle that contained 2.5 % sucrose solution and a second bottle filled with water, for 2 h. The percentage preference was calculated according to the following formula: sucrose intake percentage % = [sucrose intake / (sucrose + water intake)] plus 100 %]^75^.

### Tail-suspension test (TST)

Mice were suspended by their tails at 20 cm from ground for 6 min. The time of immobility was recorded^57^.

### Statistics and reproducibility

For statistical analysis we used two-tailed *t*-test or one-way analysis of variance (ANOVA) followed by a Tukey post hoc multiple comparison test for unequal replications using GraphPad Prism 8 software (GraphPad Software Inc., La Jolla, CA) and SPSS 24 software (International Business Machines Corp., NY, USA). The differences among multiple groups were analysed by two-way ANOVA followed by a Tukey post hoc multiple comparison test. All statistical data in the text are presented as the mean ± SD, the value of significance was set at *p* < 0.05.

### Reporting summary

Further information on research design is available in the Nature Research Reporting Summary linked to this article.

## Supplementary information

Supplementary Information

Description of Additional Supplementary Files

Supplementary Data 1

Supplementary Data 2

Reporting Summary

## Data Availability

Source data underlying the main and supplementary figures are available in Supplementary Data 1. The details of statistical analysis for all figures are available in Supplementary Data 2. The uncropped blots are available in Supplementary Information file (Supplementary Fig. 8). The data that support the findings of this study are available from the corresponding author Baoman Li upon reasonable request.
