# Supplementary material for: The neuroprotective mechanism of lithium after ischaemic stroke
Source: Commun Biol. 2022 Feb 3;5:105. doi: 10.1038/s42003-022-03051-2 (PMC8814028; doi:10.1038/s42003-022-03051-2)
Supplement: Supplementary file 1 — Supplementary Information [file 42003_2022_3051_MOESM1_ESM.pdf]

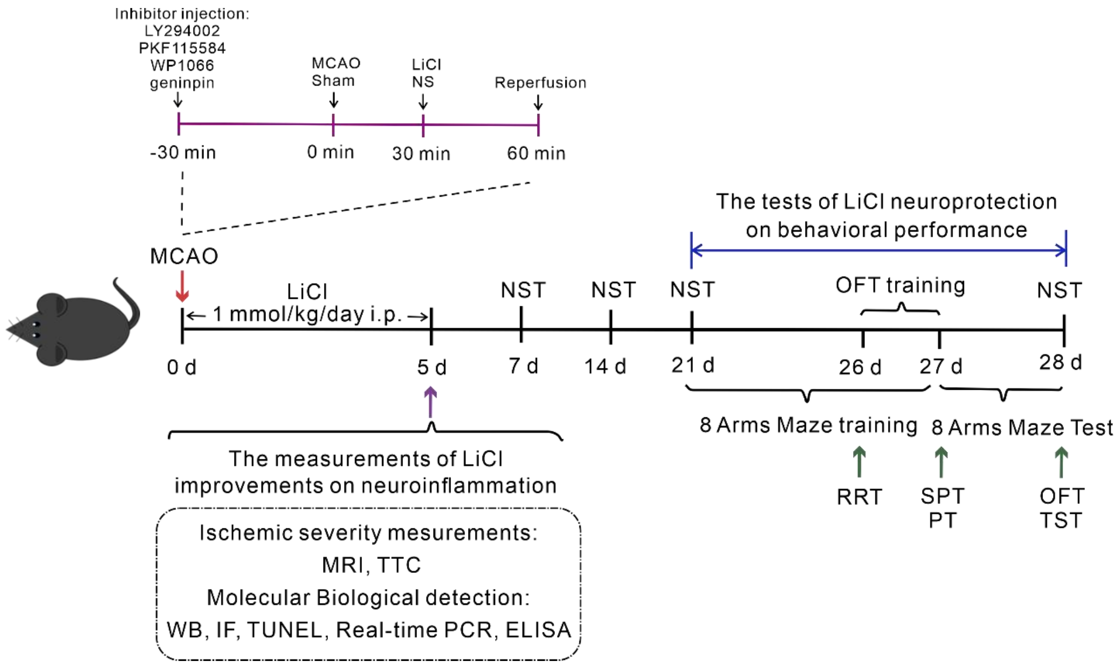

Supplementary Figure 1. Experimental design and drug treatments.
Mice were randomly separated into sham + normal solution (NS) group (control), sham + lithium chloride group (LiCl), MCAO + NS group (MCAO), MCAO + LiCl group, inhibitors + MCAO + NS group and inhibitors + MCAO + LiCl groups. In the last two groups, 30 minutes before the surgery, a randomly assigned mice were intraperitoneally injected with 12.5 mg/kg/day LY294002 (AKT inhibitor), 0.2 mg/kg/day PKF115584 (antagonist of β-catenin/TCF4 complex), 30 mg/kg/day WP1066 (STAT3 inhibitor) or 2.5 mg/kg/day geninpin (UCP2 inhibitor). All mice were subjected to MCAO or treated with sham operation for 60 minutes, at the middle point (30 minutes), 1 mmol/kg/day LiCl or normal saline (NS) were randomly intraperitoneal injected. After 60 minutes of ischaemia, the middle cerebral artery was reperfused. The injection of all reagents was repeated once a day for 5 days. Subsequently, 6 mice form each group were sacrificed by cervical dislocation for histology and biochemistry; remaining mice were used for behavioural tests as indicated.

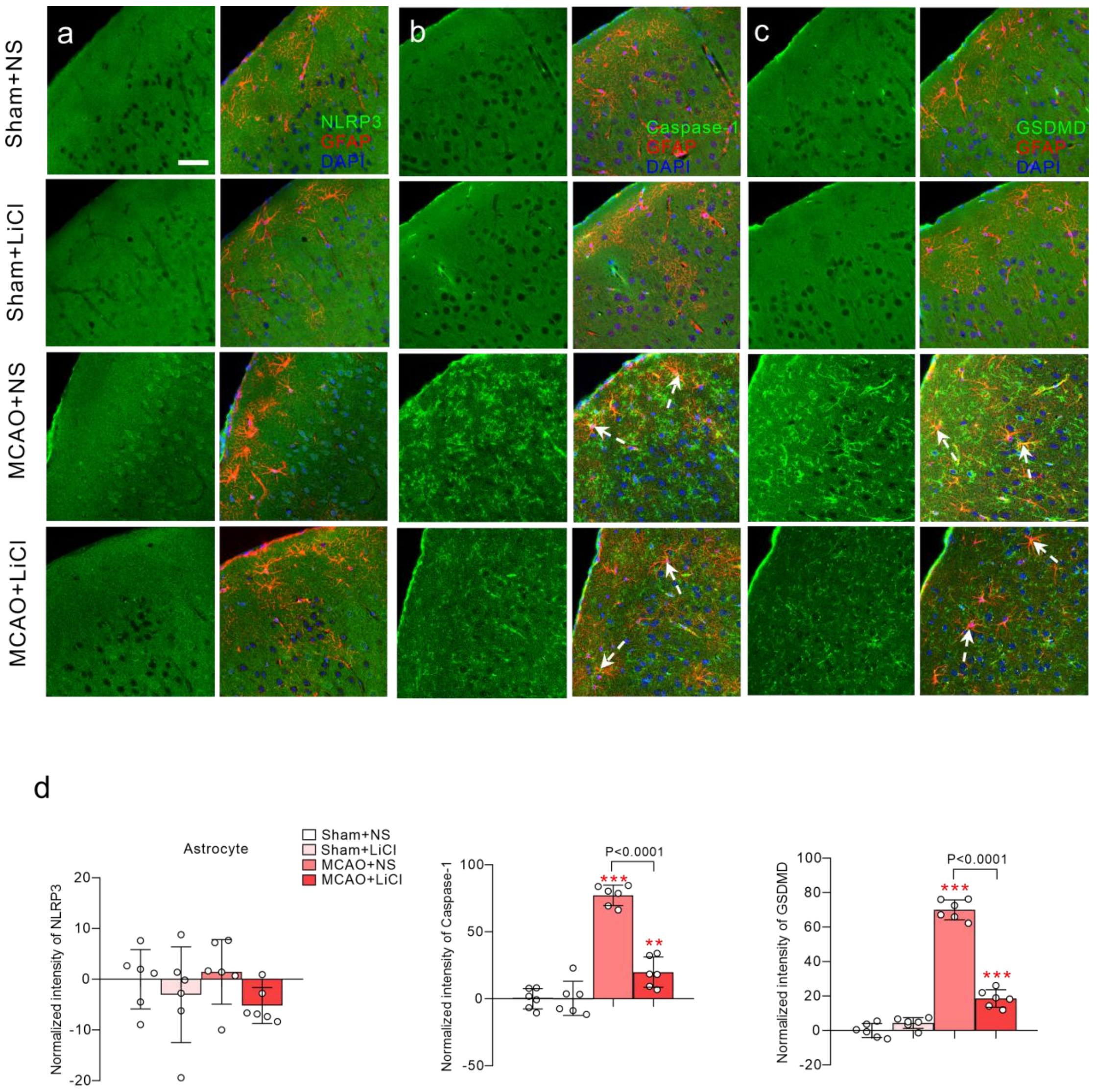

Supplementary Figure 2. LiCl suppresses astrocytic activation of NLRP3 inflammasome and the pyroptosis related GSDMD induced by MCAO.

(a - c): Green immunofluorescence of NLRP3 (a), caspase-1 (b) and GSDMD (c) co-stained with astrocytic marker GFAP (red) and nucleus marker DAPI (blue) in the injured ipsilateral cortex. Scale bar 50 µm.

(d): Immunofluorescence intensities of NLRP3, caspase-1 and GSDMD in the astrocytes of cortex were normalised to sham-NS group. The immunofluorescence intensities were normalised to control group and plotted as mean ± SD. N=6 per group. One-way ANOVA for comparisons including more than two groups; unpaired two-tailed t-test for two group comparisons. *p<0.05, **p<0.01, ***p<0.001.

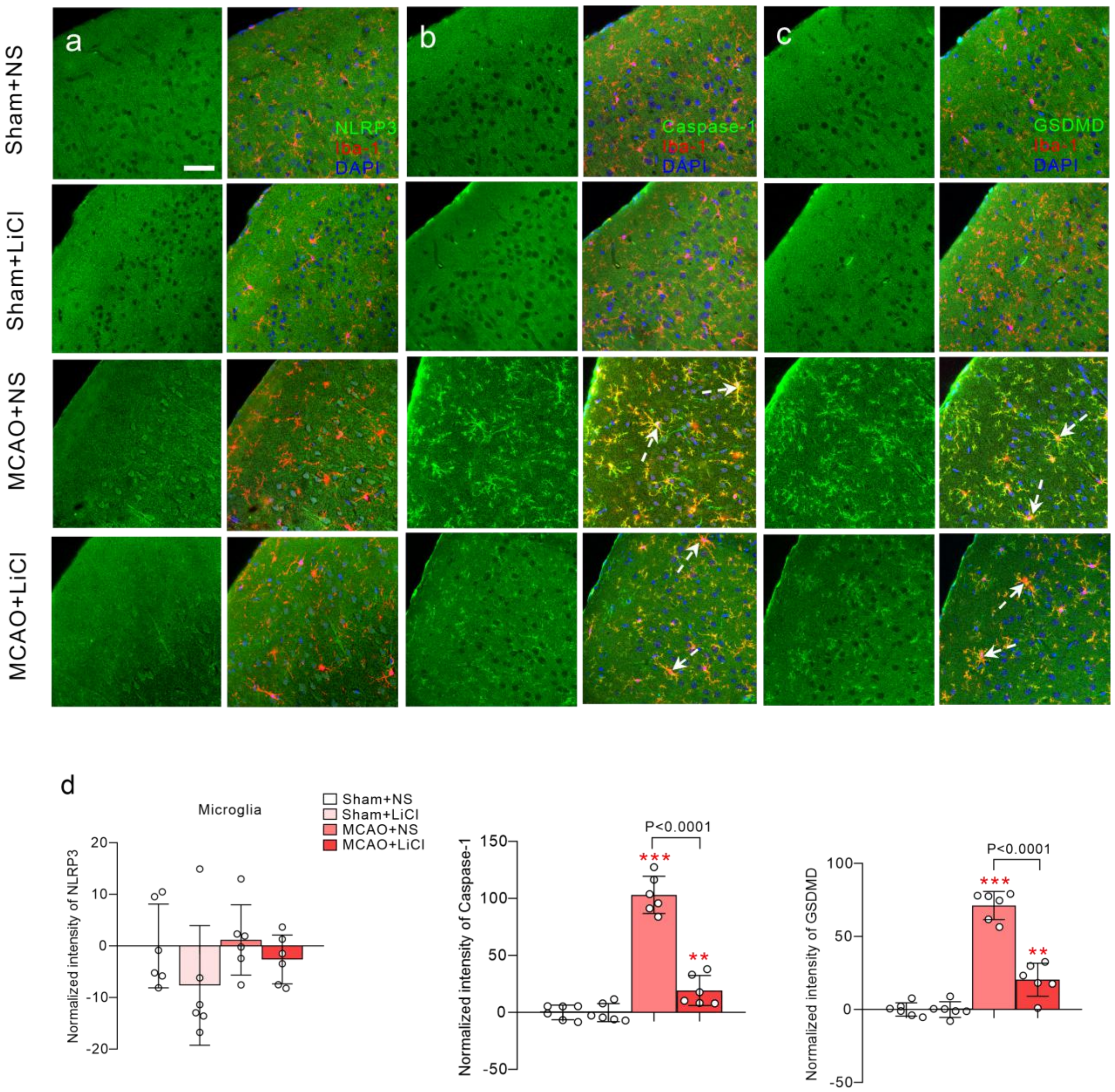

Supplementary Figure 3. LiCl suppresses microglial activation of NLRP3 inflammasome and the pyroptosis related GSDMD induced by MCAO.

(a - c): Green immunofluorescence of NLRP3 (a), caspase-1 (b) and GSDMD (c) co-stained with microglial marker Iba1 (red) and nucleus marker DAPI (blue) in the injured ipsilateral cortex. Scale bar 50 µm.

(d): Immunofluorescence intensities of NLRP3, caspase-1 and GSDMD in the microglia of cortex were normalised by sham-NS group. The immunofluorescence intensities were normalised to control group and plotted as mean ± SD. N=6 per group. One-way ANOVA for comparisons including more than two groups; unpaired two-tailed t-test for two group comparisons. *p<0.05, **p<0.01, ***p<0.001.

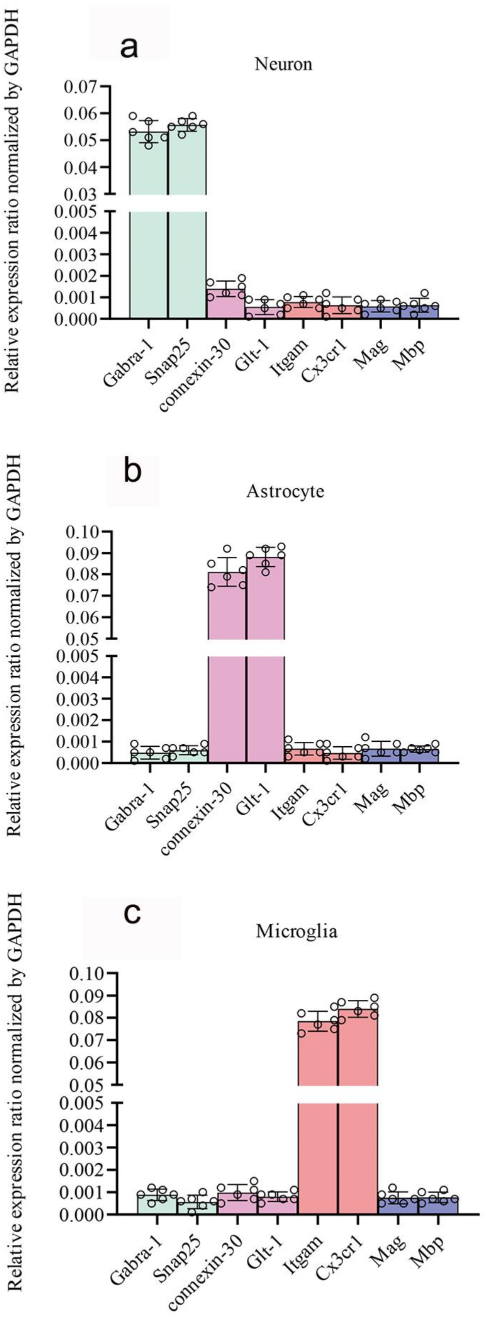

Supplementary Figure 4. The mRNA expression of cellular specific genes in neurones, astrocytes and microglia sorted by FACS.

The purity of neurons (a), astrocytes (b) and microglia (c) sorted by FACS were identified by real-time PCR. Neuronal markers (Gabra-1 and Snap25), astrocyte markers (connexin-30 and Glt-1), microglia markers (Itgam and Cx3cr1) and oligodendrocyte markers (Mag and Mbp) were measured by real-time PCR. The relative expression ratio normalized by GAPDH are presented as mean ± SD. N=6 per group.

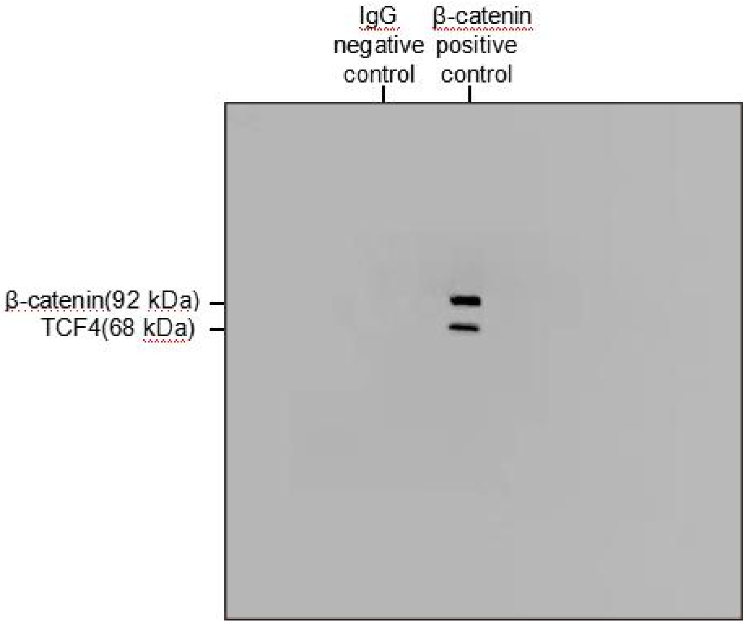

Supplementary Figure 5. The negative control for co-immunoprecipitation.
The same lysates of mice cerebral cortex were incubated with 20 μg of the mouse normal IgG (negative control) or 20 μg of β-catenin antibody (positive control), and then the protein bands of β-catenin and TCF4 were measured together in the followed western blotting.

a

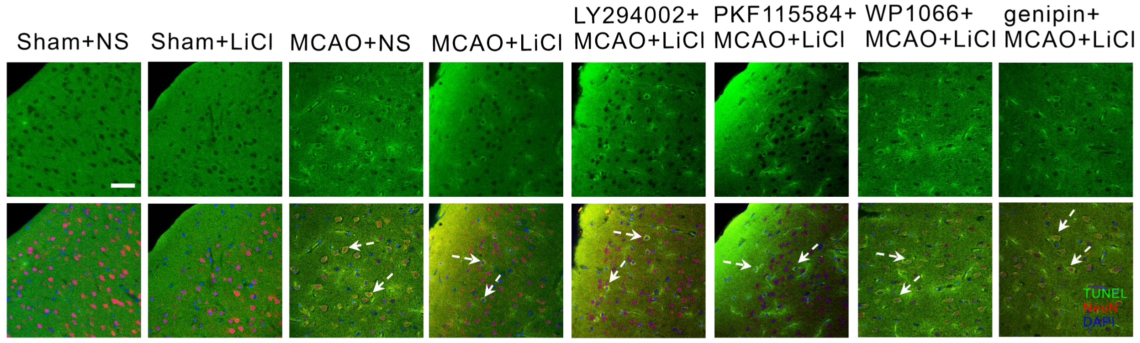

b

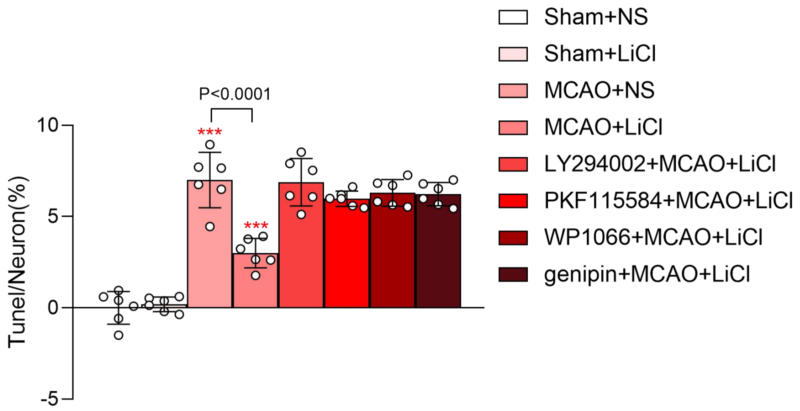

Supplementary Figure 6. Reduction of neuronal apoptosis by Li^+.
(a): TUNEL co-staining with neuronal marker NeuN and nucleus marker DAPI, and the relative ratio of Tunel-positive cells in NeuN-positive cells. Scar = 50 μm.
(b): The percentage of neuronal apoptosis were calculated and plotted as mean ± SD. N=6 per group. One-way ANOVA for comparisons including more than two groups; unpaired two-tailed t-test for two group comparisons. *p<0.05, **p<0.01, ***p<0.001.

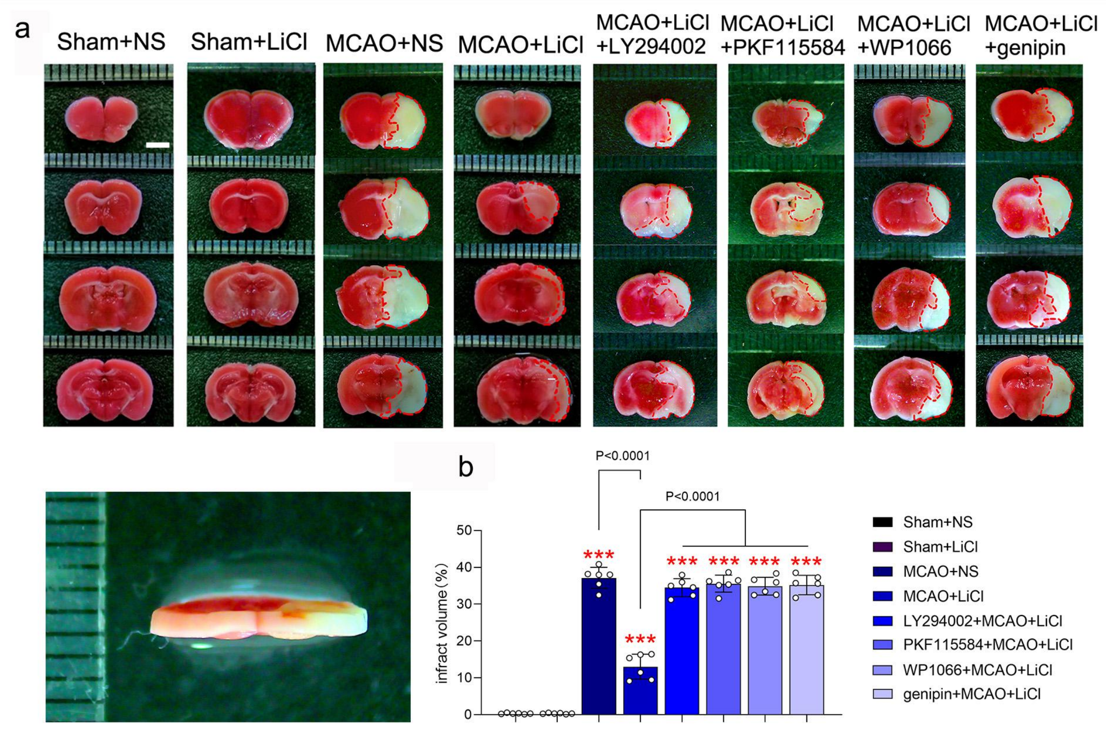

Supplementary Figure 7. Reduction of ischaemic volume by Li^{+}.

(a): TTC staining in brain slices, and the percentage of infarct volume.

(b): The percentage of ischaemic volume normalized by control (sham+NS) group were calculated and plotted as mean ± SD. N=6 per group. One-way ANOVA for comparisons including more than two groups; unpaired two-tailed t-test for two group comparisons. *p<0.05, **p<0.01, ***p<0.001.

Supplementary Figure 8. Original figures of Western Blot.

Figure 2

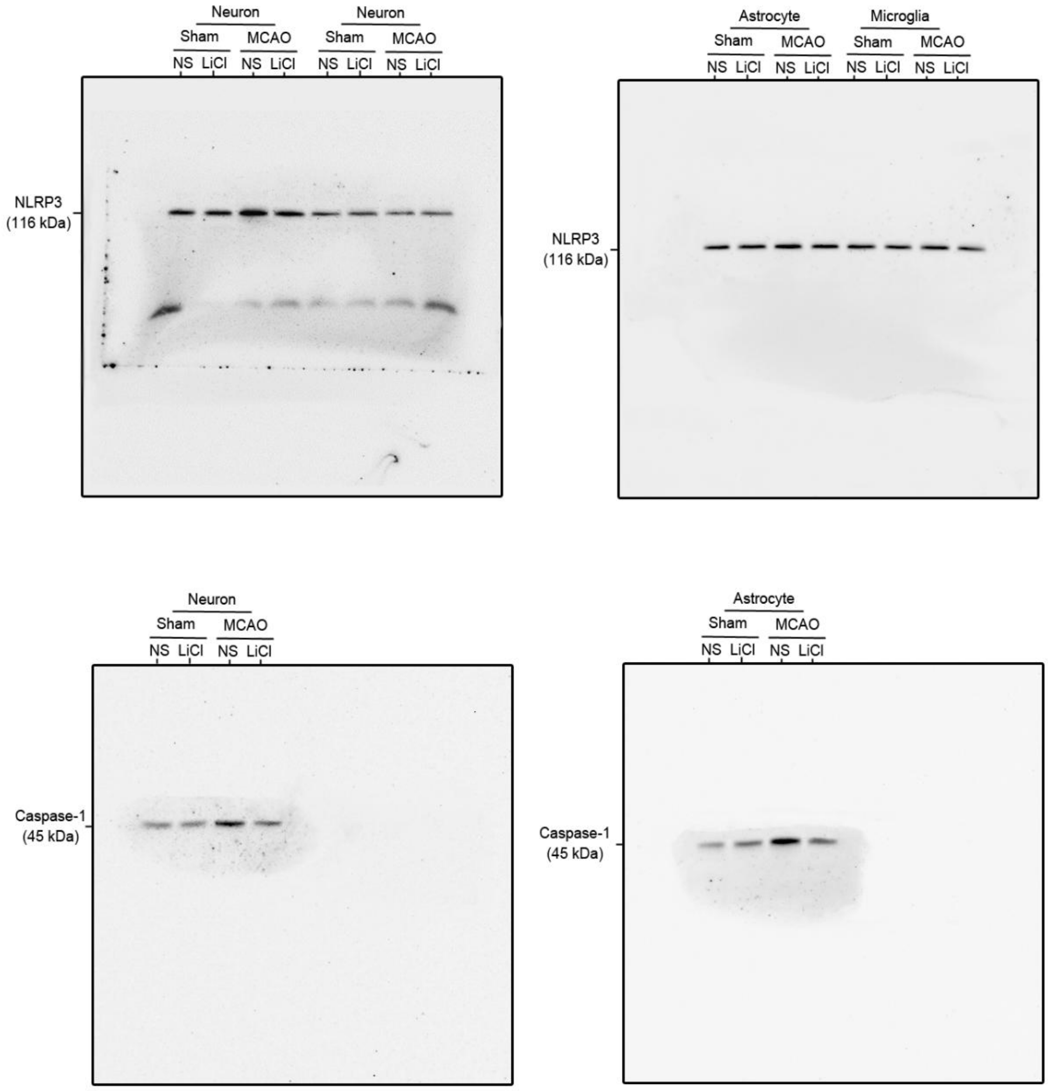

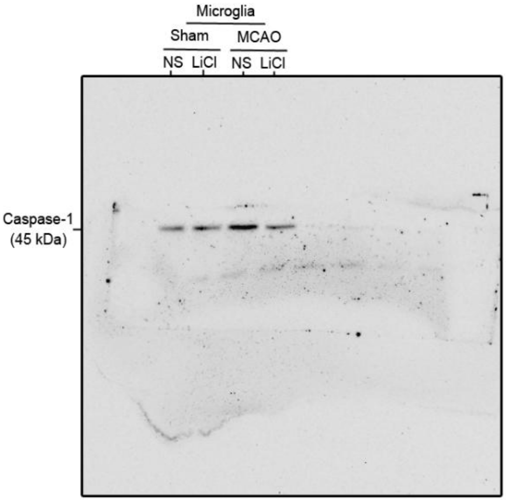
Microglia
Sham
MCAO
NS
LiCl
NS
LiCl
Caspase-1
(45 kDa)

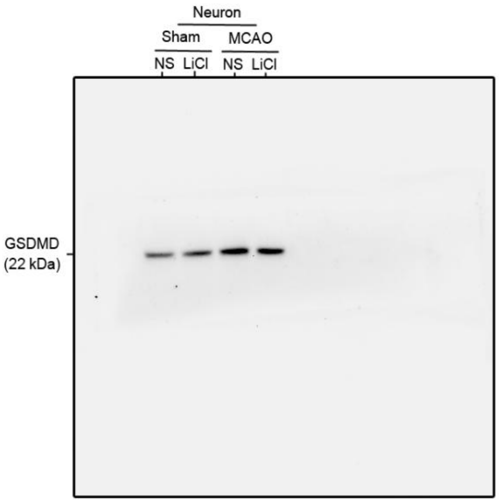
Neuron
Sham
MCAO
NS
LiCl
NS
LiCl
GSDMD
(22 kDa)

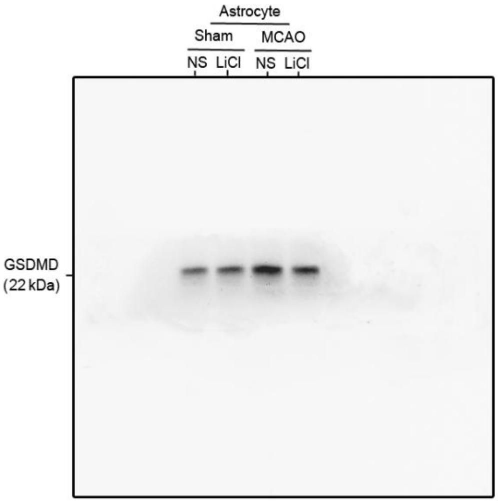
Astrocyte
Sham
MCAO
NS
LiCl
NS
LiCl
GSDMD
(22 kDa)

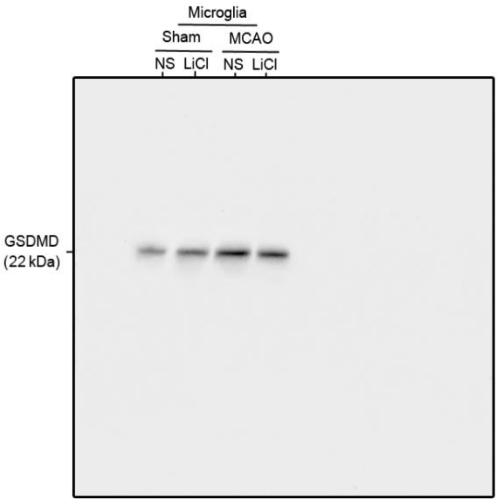
Microglia
Sham
MCAO
NS
LiCl
NS
LiCl
GSDMD
(22 kDa)

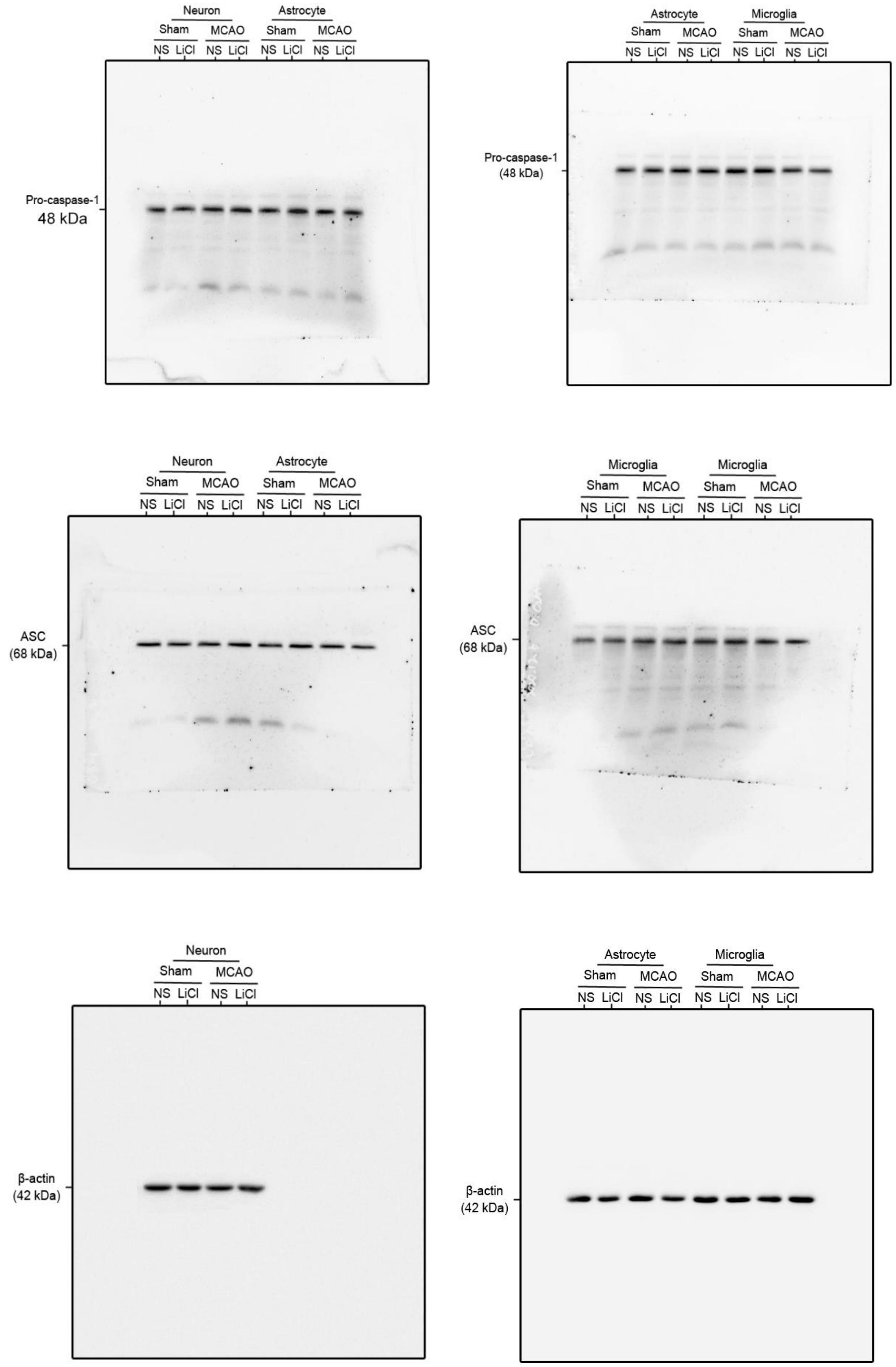
Neuron
Astrocyte
Sham
MCAO
NS
LiCl
Pro-caspase-1
48 kDa
Astrocyte
Microglia
Sham
MCAO
NS
LiCl
Pro-caspase-1
(48 kDa)
Neuron
Astrocyte
Sham
MCAO
NS
LiCl
ASC
(68 kDa)
Microglia
Microglia
Sham
MCAO
NS
LiCl
ASC
(68 kDa)
Neuron
Sham
MCAO
NS
LiCl
β-actin
(42 kDa)
Astrocyte
Microglia
Sham
MCAO
NS
LiCl
β-actin
(42 kDa)

Figure 3

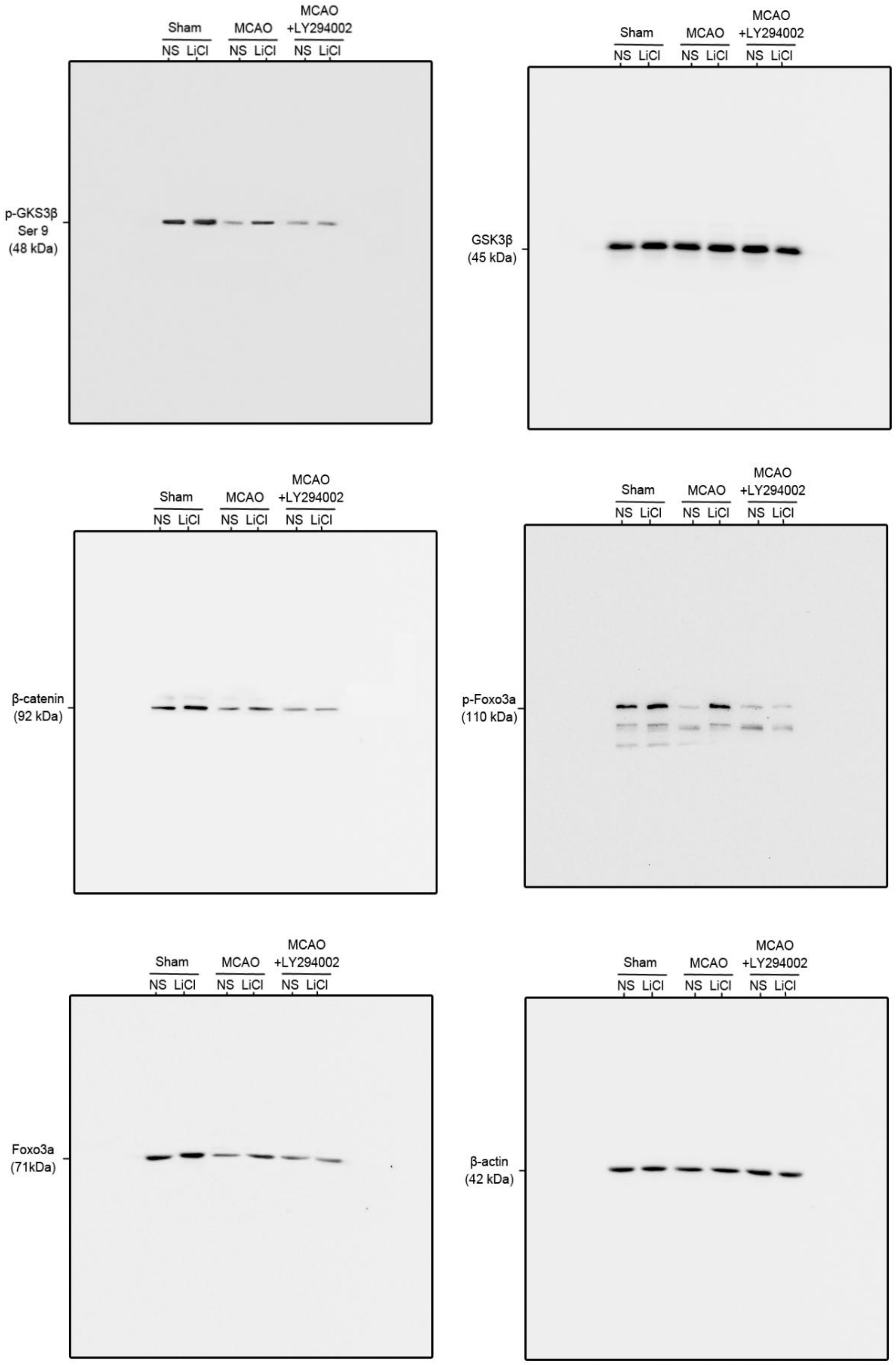

Sham
MCAO
MCAO +LY294002
NS LiCl NS LiCl NS LiCl
p-GKS3β Ser 9 (48 kDa)
GSK3β (45 kDa)
β-catenin (92 kDa)
p-Foxo3a (110 kDa)
Foxo3a (71kDa)
β-actin (42 kDa)

Figure 4

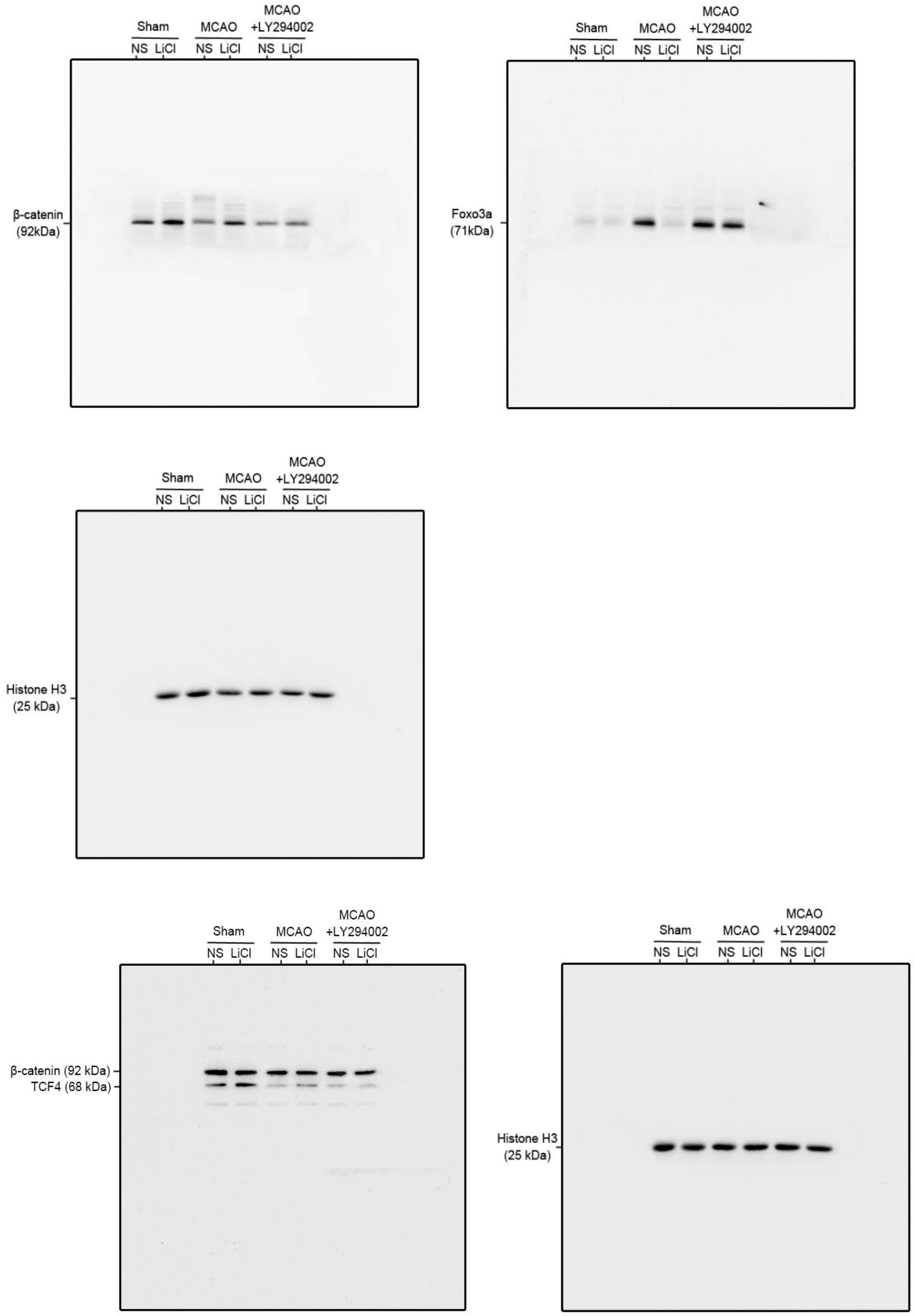
Sham
MCAO
MCAO +LY294002
NS LiCl NS LiCl NS LiCl
β-catenin (92kDa)
Foxo3a (71kDa)
Histone H3 (25 kDa)
β-catenin (92 kDa)
TCF4 (68 kDa)
Histone H3 (25 kDa)

Figure 5

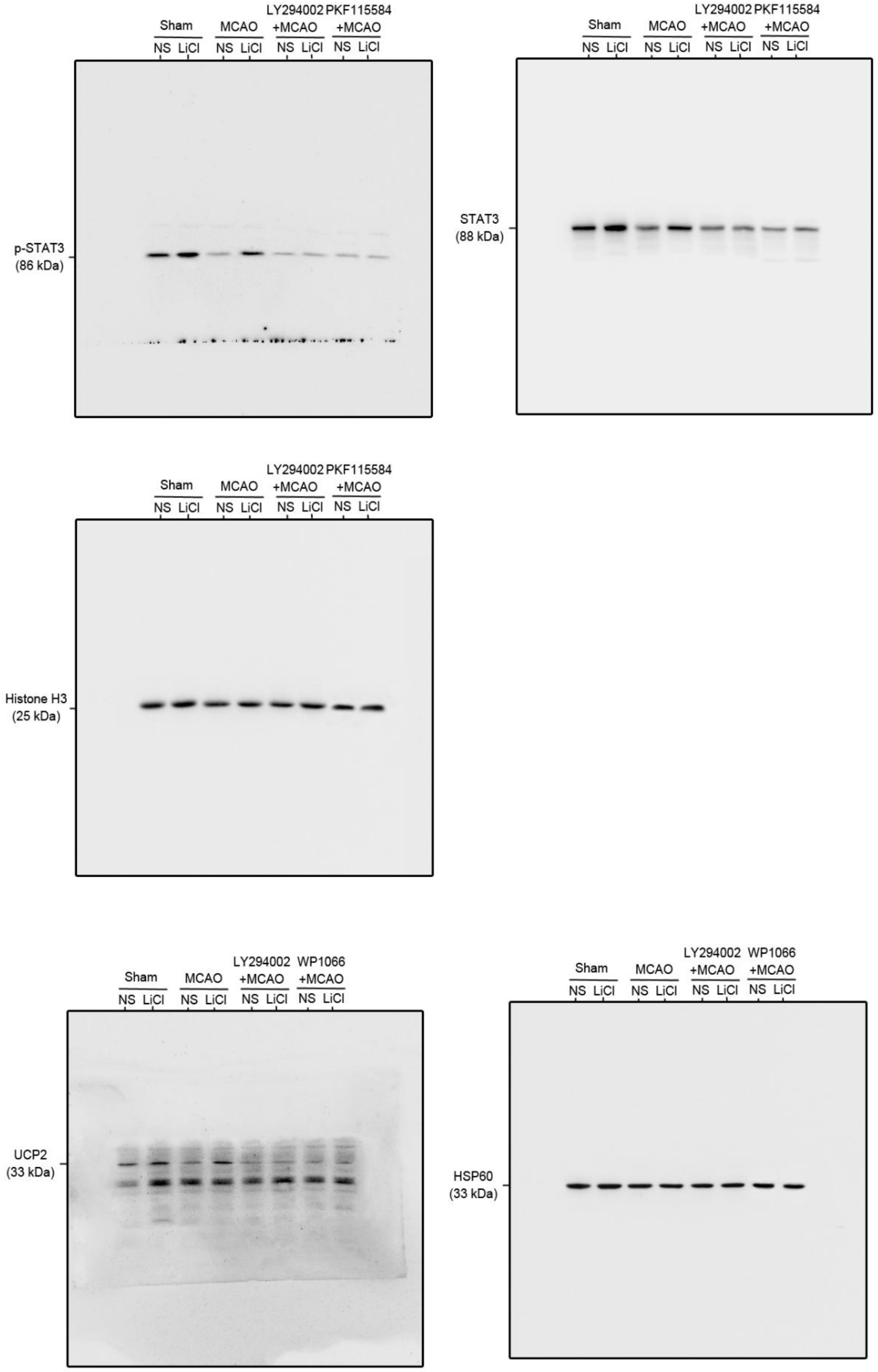
Sham
MCAO
LY294002 +MCAO
PKF115584 +MCAO
NS LiCl NS LiCl NS LiCl NS LiCl
p-STAT3 (86 kDa)
STAT3 (88 kDa)
Histone H3 (25 kDa)
WP1066 +MCAO
UCP2 (33 kDa)
HSP60 (33 kDa)

Figure 6

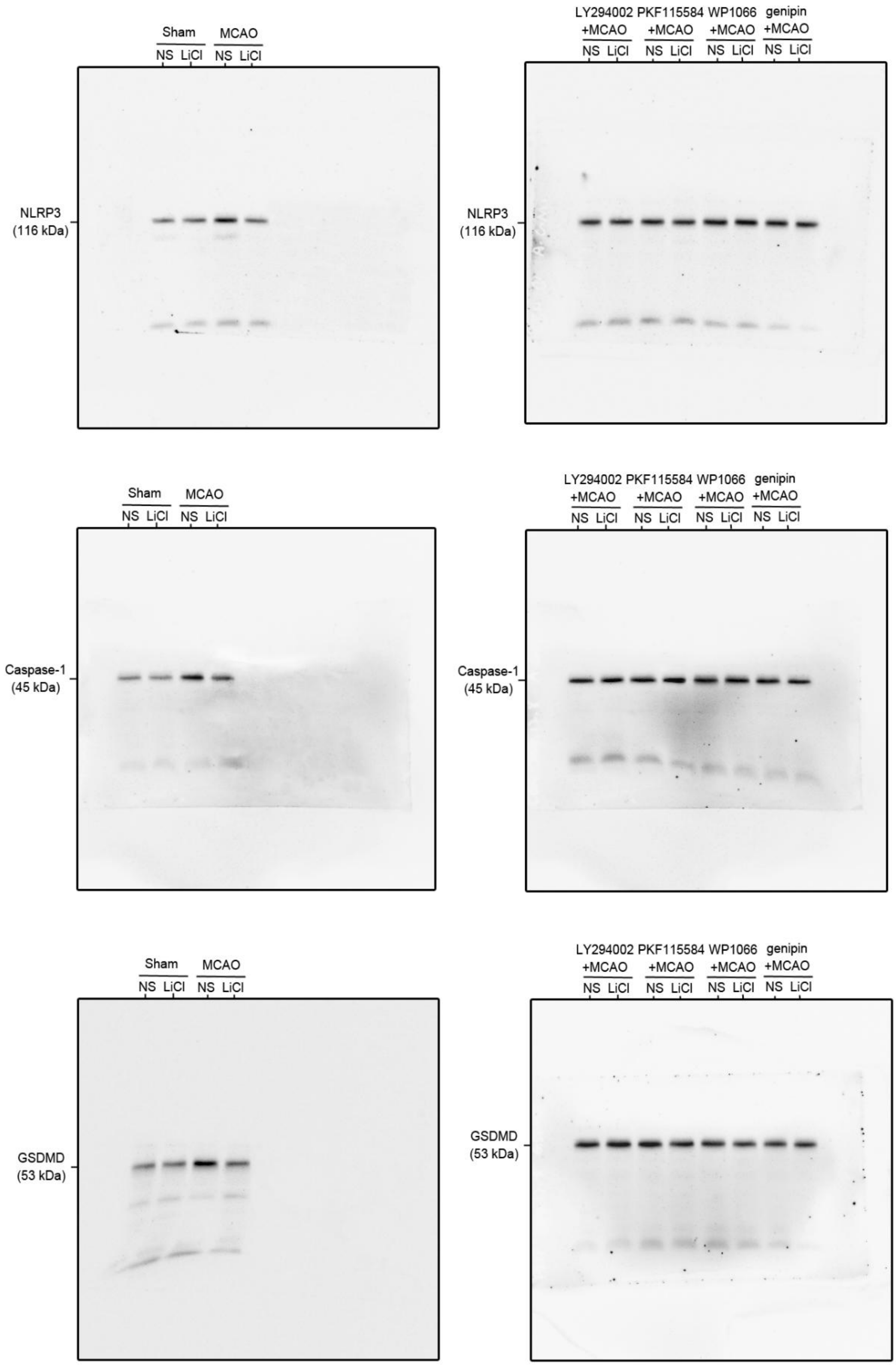
Sham
MCAO
NS LiCl NS LiCl
NLRP3
(116 kDa)
LY294002 +MCAO
PKF115584 +MCAO
WP1066 +MCAO
genipin +MCAO
NS LiCl NS LiCl NS LiCl NS LiCl
NLRP3
(116 kDa)
Sham
MCAO
NS LiCl NS LiCl
Caspase-1
(45 kDa)
LY294002 +MCAO
PKF115584 +MCAO
WP1066 +MCAO
genipin +MCAO
NS LiCl NS LiCl NS LiCl NS LiCl
Caspase-1
(45 kDa)
Sham
MCAO
NS LiCl NS LiCl
GSDMD
(53 kDa)
LY294002 +MCAO
PKF115584 +MCAO
WP1066 +MCAO
genipin +MCAO
NS LiCl NS LiCl NS LiCl NS LiCl
GSDMD
(53 kDa)

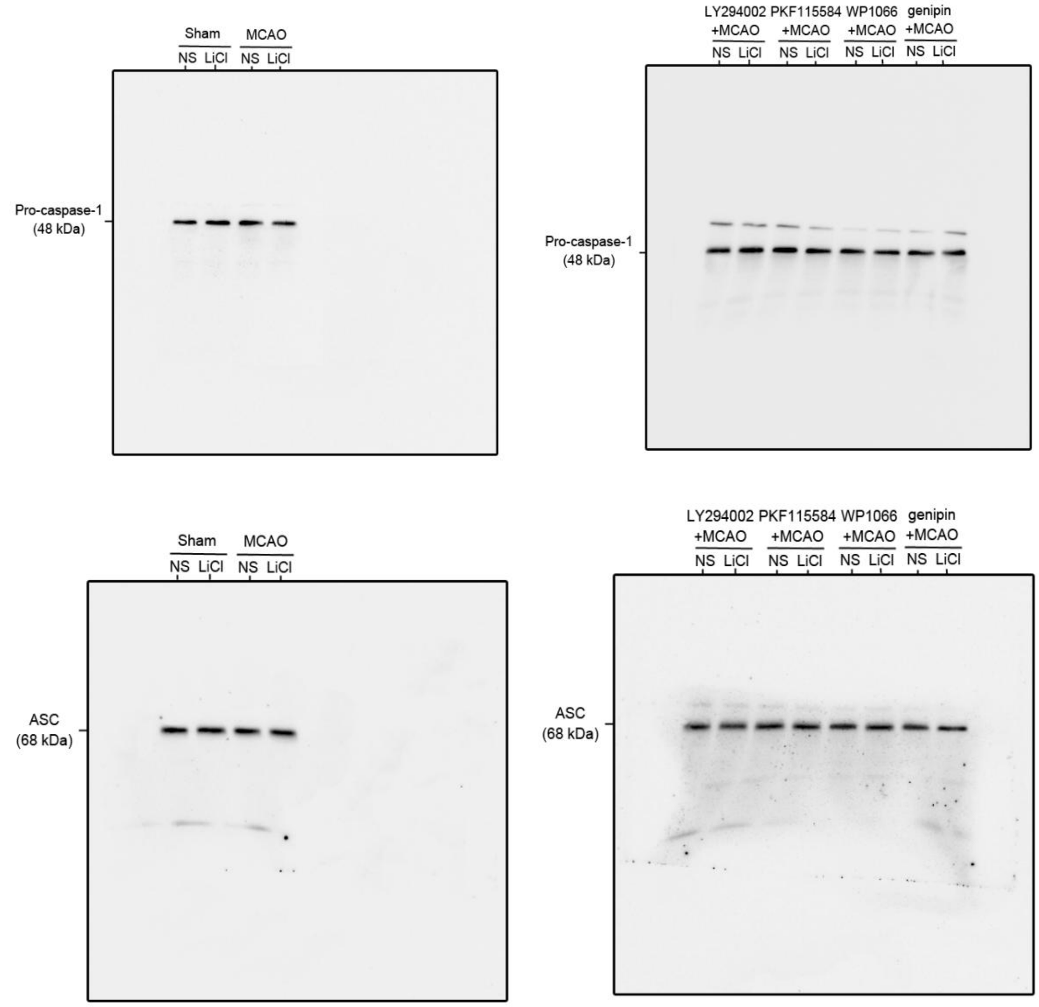
Sham
MCAO
NS LiCl NS LiCl
Pro-caspase-1
(48 kDa)
LY294002 +MCAO
PKF115584 +MCAO
WP1066 +MCAO
genipin +MCAO
NS LiCl NS LiCl NS LiCl NS LiCl
Pro-caspase-1
(48 kDa)
Sham
MCAO
NS LiCl NS LiCl
ASC
(68 kDa)
LY294002 +MCAO
PKF115584 +MCAO
WP1066 +MCAO
genipin +MCAO
NS LiCl NS LiCl NS LiCl NS LiCl
ASC
(68 kDa)

Figure S5

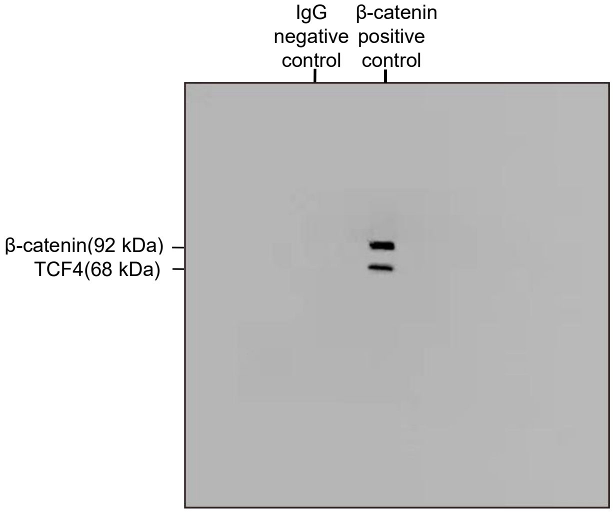
IgG negative control
β-catenin positive control
β-catenin(92 kDa)
TCF4(68 kDa)
