# Supplementary material for: The neuroprotective mechanism of lithium after ischaemic stroke
Source: Commun Biol. 2022 Feb 3;5:105. doi: 10.1038/s42003-022-03051-2 (PMC8814028; doi:10.1038/s42003-022-03051-2)
Supplement: Supplementary file 2 — Description of Additional Supplementary Files [file 42003_2022_3051_MOESM2_ESM.pdf]

Description of Additional Supplementary Files

File name: Supplementary Data 1
Description: Source data of all figures.

File name: Supplementary Data 2
Description: Statistical analysis for all figures.
