# Supplementary material for: The neuroprotective mechanism of lithium after ischaemic stroke
Source: Commun Biol. 2022 Feb 3;5:105. doi: 10.1038/s42003-022-03051-2 (PMC8814028; doi:10.1038/s42003-022-03051-2)
Supplement: Supplementary file 5 — Reporting Summary [file 42003_2022_3051_MOESM5_ESM.pdf]

nature portfolio

Corresponding author(s): Baoman Li

Last updated by author(s): Dec 5, 2021

Reporting Summary

Nature Portfolio wishes to improve the reproducibility of the work that we publish. This form provides structure for consistency and transparency in reporting. For further information on Nature Portfolio policies, see our Editorial Policies and the Editorial Policy Checklist.

Statistics

For all statistical analyses, confirm that the following items are present in the figure legend, table legend, main text, or Methods section.

n/a	Confirmed	
☐	☒	The exact sample size (*n*) for each experimental group/condition, given as a discrete number and unit of measurement
☐	☒	A statement on whether measurements were taken from distinct samples or whether the same sample was measured repeatedly
☐	☒	The statistical test(s) used AND whether they are one- or two-sided<br>*Only common tests should be described solely by name; describe more complex techniques in the Methods section.*
☐	☒	A description of all covariates tested
☐	☒	A description of any assumptions or corrections, such as tests of normality and adjustment for multiple comparisons
☐	☒	A full description of the statistical parameters including central tendency (e.g. means) or other basic estimates (e.g. regression coefficient) AND variation (e.g. standard deviation) or associated estimates of uncertainty (e.g. confidence intervals)
☐	☒	For null hypothesis testing, the test statistic (e.g. *F*, *t*, *r*) with confidence intervals, effect sizes, degrees of freedom and *P* value noted<br>*Give P values as exact values whenever suitable.*
☒	☐	For Bayesian analysis, information on the choice of priors and Markov chain Monte Carlo settings
☐	☒	For hierarchical and complex designs, identification of the appropriate level for tests and full reporting of outcomes
☐	☒	Estimates of effect sizes (e.g. Cohen's *d*, Pearson's *r*), indicating how they were calculated

Our web collection on statistics for biologists contains articles on many of the points above.

Software and code

Policy information about availability of computer code

Data collection	One-way analysis of variance (ANOVA) followed by a Tukey's or Dunnett's post hoc multiple comparison test for unequal replications using GraphPad Prism 8 software (GraphPad Software Inc., La Jolla, CA) and SPSS 24 software (International Business Machines Corp., NY, USA).The differences among multiple groups were analyzed by two-way ANOVA followed by a Tukey post hoc multiple comparison test.
Data analysis	All data were expressed as mean and standard deviation (SD) and were analyzed two-way ANOVA followed by a Tukey post hoc multiple comparison test with a value of <0.05 considered to be statistically significance.

For manuscripts utilizing custom algorithms or software that are central to the research but not yet described in published literature, software must be made available to editors and reviewers. We strongly encourage code deposition in a community repository (e.g. GitHub). See the Nature Portfolio guidelines for submitting code & software for further information.

Data

Policy information about availability of data

All manuscripts must include a data availability statement. This statement should provide the following information, where applicable:

- Accession codes, unique identifiers, or web links for publicly available datasets
- A description of any restrictions on data availability
- For clinical datasets or third party data, please ensure that the statement adheres to our policy

The data that support the findings of this study are available from the corresponding author upon reasonable request.

Field-specific reporting

Please select the one below that is the best fit for your research. If you are not sure, read the appropriate sections before making your selection.

☒ Life sciences ☐ Behavioural & social sciences ☐ Ecological, evolutionary & environmental sciences

For a reference copy of the document with all sections, see nature.com/documents/nr-reporting-summary-flat.pdf

Life sciences study design

All studies must disclose on these points even when the disclosure is negative.

Sample size	The number of samples in each group of immunofluorescence, co-immunoprecipitation, western blotting, real-time PCR, ROS detection, ELISA assays, TUNEL measurements, infarct volume measurement, magnetic resonance imaging and other behavioural tests(NST, RRT, PT, OFT, 8 arms maze test, SPT, TST) are 6.
Data exclusions	No data are excluded.
Replication	We confirm that the measurements in this study were successful replicated.
Randomization	The experimental animals were randomly assigned to different experimental groups with a random number table.
Blinding	Immunofluorescence, co-immunoprecipitation, western blotting, real-time PCR, ROS detection, ELISA assays, TUNEL measurements, infarct volume measurement, magnetic resonance imaging and other behavioural tests were performed by investigators blinded to the experimental conditions.

Reporting for specific materials, systems and methods

We require information from authors about some types of materials, experimental systems and methods used in many studies. Here, indicate whether each material, system or method listed is relevant to your study. If you are not sure if a list item applies to your research, read the appropriate section before selecting a response.

Materials & experimental systems

n/a	Involved in the study	
☐	☒	Antibodies
☒	☐	Eukaryotic cell lines
☒	☐	Palaeontology and archaeology
☐	☒	Animals and other organisms
☒	☐	Human research participants
☒	☐	Clinical data
☒	☐	Dual use research of concern

Methods

n/a	Involved in the study	
☒	☐	ChIP-seq
☒	☐	Flow cytometry
☐	☒	MRI-based neuroimaging

Antibodies

Antibodies used	Primary antibody of ASC (sc-365611), caspase-1(sc-56036), β-catenin (sc-59737) , GSDMD (sc-393656) and p-STAT3 (sc-81523) were purchase from Santa Cruz Biotechnology (Santa Cruz, CA, USA). Primary antibody of NLPR3 (ab214185), pro-caspase-1 (ab179515), GFAP (ab48050), Iba-1 antibody used for FACS (ab178846) and secondary antibody Alexa Fluor 555 goat anti rabbit (ab150074) were purchased from Abcam (Cambridge, MA, USA). Primary antibody of Foxo3a (720128), p-Foxo3a antibody (PA5-37578), p-GSK3β Ser9 (MA5-14873), UCP2 (PA5-80203), NeuN (PA5-78693) and Alexa Fluor-conjugated 488 secondary antibody used for FACS (A-11001) were purchased from Thermo Fisher Scientific (Waltham, MA USA). Primary antibody of GSK3β(22104-1-AP), TCF4 (22337-1-AP) and STAT3 (10253-2-AP) were purchased from Proteintech (Wuhan, Hubei, China). Primary antibody of β-actin (E021020), Histone H3 (E021130), HSP60 (A200659) and secondary antibody HRP-labelled Goat anti Mouse (E030110) and HRP-labeled Goat anti Rabbit (E030120) were purchased from Earthox (Millbrae, CA, USA). Primary antibody of Iba-1 used for immunofluorescence (019-19741) was purchased from Wako Chemicals (USA) and Alexa Fluor-conjugated 488/555 secondary antibody (A21202, A31570) were from Gibco Life Technology Invitrogen (Grand Island, NY, USA).
Validation	All primary antibodies all have mouse species reactivity and could be applied in western-blotting, immuno-fluorescence or co-immunoprecipitation.

Animals and other organisms

Policy information about studies involving animals; ARRIVE guidelines recommended for reporting animal research

Laboratory animals	The C57BL/6 mice(#000664), FVB/N-Tg(GFAP-eGFP)14Mes/J(#003257) and B6.Cg-Tg(Thy1-YFP)HJrs/J (#003782) transgenic mice were all purchased from the Jackson Laboratory (Bar Harbor, ME, USA). For all the experiments mice were male and 12-16 weeks old.
Wild animals	The study does not involved wild animals.
Field-collected samples	The animals were raised in standard housing conditions (22 ± 1?; light/dark cycle of 12/12h), with water and food available ad libitum.
Ethics oversight	All experiments were performed in accordance with the US National Institutes of Health Guide for the Care and Use of Laboratory Animals (NIH Publication No. 8023) and its 1978 revision, and all experimental protocols were approved by the Institutional Animal Care and Use Committee of China Medical University, No. [2020]102.

Note that full information on the approval of the study protocol must also be provided in the manuscript.

Magnetic resonance imaging

Experimental design

Design type	In vivo MRI experiments.
Design specifications	The brains in MCAO mice treated with NS or Li+ were observed by MRI, separately.
Behavioral performance measures	n/a

Acquisition

Imaging type(s)	Structral
Field strength	3.0T
Sequence & imaging parameters	A fast-spin-echo (FSE) T2-weighted sequence. Echo time/repetition time=85.46/4620 ms, field of view=40mm×40mm, 10 slices with 1.5mm slice thickness, in-plane resolution of 512×512 pixel, and an imaging time of 12minutes.
Area of acquisition	A whole brain scan

Diffusion MRI ☐ Used ☒ Not used

Preprocessing

Preprocessing software	Signa 3.0T HDxt system (GE Healthcare, Fairfield, Connecticut, USA).
Normalization	n/a
Normalization template	n/a
Noise and artifact removal	The average respiratory rate was 90 ± 15 breaths/min, and the average heart rate was 450 ± 20 bpm.
Volume censoring	n/a

Statistical modeling & inference

Model type and settings	n/a
Effect(s) tested	n/a

Specify type of analysis: ☒ Whole brain ☐ ROI-based ☐ Both

Statistic type for inference (See Eklund et al. 2016)	n/a
Correction	n/a

Models & analysis

n/a	Involved in the study
☒	☐ Functional and/or effective connectivity
☒	☐ Graph analysis
☒	☐ Multivariate modeling or predictive analysis
